# A glimpse into the genetic diversity of the Peruvian seafood sector: Unveiling species substitution, mislabeling and trade of threatened species

**DOI:** 10.1371/journal.pone.0206596

**Published:** 2018-11-16

**Authors:** Alan Marín, José Serna, Christian Robles, Beder Ramírez, Lorenzo E. Reyes-Flores, Eliana Zelada-Mázmela, Giovanna Sotil, Ruben Alfaro

**Affiliations:** 1 Biodes Laboratorios Soluciones Integrales S.C.R.L., Tumbes, Perú; 2 Laboratorio Costero de Tumbes, Instituto del Mar del Perú-IMARPE, Tumbes, Perú; 3 Laboratorio de Genética, Fisiología y Reproducción, Facultad de Ciencias, Universidad Nacional del Santa, Chimbote, Perú; 4 Laboratorio de Genética Molecular, Instituto del Mar del Perú-IMARPE, Lima, Perú; 5 Laboratorio de Biología Molecular, Facultad de Ciencias de la Salud, Universidad Nacional de Tumbes, Tumbes, Perú; Chinese Academy of Medical Sciences and Peking Union Medical College, CHINA

## Abstract

Peru is one of the world’s leading fishing nations and its seafood industry relies on the trade of a vast variety of aquatic resources, playing a key role in the country’s socio-economic development. DNA barcoding has become of paramount importance for systematics, conservation, and seafood traceability, complementing or even surpassing conventional identification methods when target organisms show similar morphology during the early life stages, have recently diverged, or have undergone processing. Aiming to increase our knowledge of the species diversity available across the Peruvian supply chain (from fish landing sites to markets and restaurants), we applied full and mini-barcoding approaches targeting three mitochondrial genes (COI, 16S, and 12S) and the control region to identify samples purchased at retailers from six departments along the north-central Peruvian coast. DNA barcodes from 131 samples were assigned to 55 species (plus five genus-level taxa) comprising 47 families, 24 orders, and six classes including Actinopterygii (45.03%), Chondrichthyes (36.64%), Bivalvia (6.87%), Cephalopoda (6.11%), Malacostraca (3.82%), and Gastropoda (1.53%). The identified samples included commercially important pelagic (anchovy, bonito, dolphinfish) and demersal (hake, smooth-hound, Peruvian rock seabass, croaker) fish species. Our results unveiled the marketing of protected and threatened species such as whale shark, Atlantic white marlin, smooth hammerhead (some specimens collected during closed season), shortfin mako, and pelagic thresher sharks. A total of 35 samples (26.72%) were mislabeled, including tilapia labeled as wild marine fish, dolphinfish and hake labeled as grouper, and different shark species sold as “smooth-hounds”. The present study highlights the necessity of implementing traceability and monitoring programs along the entire seafood supply chain using molecular tools to enhance sustainability efforts and ensure consumer choice.

## Introduction

Peru is a major fishing country with rich marine biodiversity. About 1070 fish [1], 1018 molluscan [2], and 320 crustacean species [3] have been described from the Peruvian marine ecosystem, which is dominated by the cold nutrient-rich waters of the Humboldt Current [4]. The highly productive Peruvian upwelling system supports not only the world’s largest fishery for Peruvian anchovy (*Engraulis ringens*) [5, 6], but also other important planktivorous fish species (e.g., jack mackerel *Trachurus murphyi*) and their predators (e.g., bonito *Sarda chiliensis*, dolphinfish *Coryphaena hippurus*), which are valuable artisanal fishery resources [4]. The fishery sector plays a key role in the nation’s socio-economic growth with most artisanal production consumed directly through local markets [4]. In 2016, Peru was the fifth biggest producer of marine capture fisheries in the world, with a total production of 3.7 million tonnes [7]. Most (75%) of that production was due to anchovy catches, but high catches of other species such as Pacific chub mackerel (*Scomber japonicus*), jumbo flying squid (*Dosidicus gigas*), and South Pacific hake (*Merluccius gayi*), were also reported [8].

Furthermore, Peru has favorable conditions for fishery and aquaculture activities considering its 3080 km coastline, and 12000 lakes and lagoons [6, 9]. Peruvian aquaculture production makes up only 2% of the total seafood sector [9], and the main species include whiteleg shrimp (*Penaeus vannamei*), Peruvian scallop (*Argopecten purpuratus*), rainbow trout (*Oncorhynchus mykiss*), tilapia (*Oreochromis niloticus*), and Amazon fish paiche (*Arapaima gigas*) [9]. Fish consumption in Peru has increased in recent years. According to FAO statistics [10], the average annual per capita consumption of fish during 2013–15 was 21.8 kg, which was the highest in Latin America and the Caribbean. This increase was due in part to fish consumption campaigns launched by the Peruvian government [11]. The significant increase of fish consumption added to the depletion of marine resources and a high demand for quality seafood products, partially due to the growing number of Peruvian seafood restaurants (locally known as “*cebicherias*”), make this country’s sector a fertile area for mislabeling of species that are expensive, scarce or out of season.

Seafood fraud and species substitutions occur regularly in this sector and represent a global issue. Proper identification of food species is now a main concern not only for governments and companies, but also for consumers due to economic, regulatory, health and religious reasons [12]. However, conventional fish identification methods, based on morphological traits using field guides and taxonomic keys, may lead to misidentification when applied to morphologically similar or recently diverged species and can be utilized only when the full body is available. Mislabeling can happen at any point in the supply chain, from fisher to retailer; thus, determining how substitutions occur is complicated [13]. In a comprehensive analysis by Pardo et al. [14], which included 51 peer-reviewed seafood articles published from 2010 to 2015 and comprising 4500 samples, the average rate of reported misdescriptions was 30%. This is where the discriminatory power of DNA based tools can be successfully applied for seafood authentication, even if all morphological characters are gone after processing and cooking.

The most frequently used genetic marker for metazoan identification through DNA barcoding is a partial fragment (∼650 bp) located at the 5ʹ end of the mitochondrial cytochrome c oxidase subunit I (COI) gene. It has been successfully used to correctly identify different fresh and processed seafood samples [15]. Furthermore, in October 2011, the United States Food and Drug Administration (FDA) formally adopted DNA barcoding as the primary method for seafood identification [16]. The universal primers designed by Folmer et al. [17] are one of the most commonly used for the amplification of the “barcode COI region”; however these have failed to amplify PCR products from different marine organisms such as fishes, crabs, echinoderms, decapods, and scallops [18] (and references therein). Therefore new barcoding primer sets targeting more specific groups [19], as well as alternative mitochondrial genes such as cytochrome b, 12S rRNA, and 16S rRNA have been tested in different marine organisms [20, 21]. Another problem that challenges the amplification of full-length DNA barcoding fragment size (∼650 bp) is when dealing with samples that have been through extreme conditions such as high temperature and pressure in canning or cooking processes. Heat exposure and high pressure degrade large molecular weight genomic DNA to shorter size fragments mainly through enzymatic degradation, depurination, and hydrolysis, and sometimes highly degraded samples might display breaks or artifactual mutations [22]. To overcome this issue, shorter (100–200 bp) PCR fragments within the full-length barcode region (known as mini-barcoding markers) have proved to be an effective species identification tool when using degraded target DNA [23].

In spite of the increasing need to enforce regulations aiming for sustainable seafood industry and effective control of the trade of endangered species, few initiatives have been undertaken to evaluate the utility of molecular markers for authenticating products available from seafood retailers in South America. Most of those studies have surveyed single groups of Amazonian and Atlantic fish species from Brazil, including catfish and sawfish from supermarkets and fish markets [24, 25], Amazonian fish from local harbors and markets [26, 27], croaker filets from supplier companies [28], characiforms from street markets [29], and sharks from supermarkets [30]. From the South Pacific coast, reports include studies on Chilean species of commercial mollusks [31], commercial crabs from local markets [32], and salmon from supermarkets [33], while sharks from Peruvian fish landing sites [34] have also been reported.

Considering the scarcity of data regarding the authentication of seafood products in the Peruvian market and aiming to increase our knowledge of the species diversity along the supply chain from fish landing sites to markets and restaurants, this study assessed the utility of full and mini-barcoding markers for identifying a variety of local and imported fish and shellfish products in the seafood sector. Additionally, we evaluated the accuracy of fish labels and described the conservation status and current regulatory framework related to the threatened species detected in this study.

## Materials and methods

### Sample collection

A total of 143 national and imported seafood samples were collected from July 2016 to March 2018, covering a wide range of presentations including fresh, refrigerated, frozen, canned, dried, cooked, packed, dehydrated, marinated, fish burger, and fish roe. They included 48 samples from restaurants (RT), 29 from supermarket chains (SMC), 24 from markets (MK), 22 from fish landing sites (FLS), 12 from multimarket (MM), seven from wholesale fish market (WFM), and one from a grocery store (GS). Samples were collected along the north-central Peruvian coast in different cities from six departments namely Tumbes (TU, n = 26), Piura (PI, n = 3), Lambayeque (LA, n = 5), La Libertad (LL, n = 48), Ancash (AN, n = 25), and Lima (LI, n = 36). Sampling localities were chosen due to the more diverse marine ichthyofauna present in the north than in the south [6]. Packages and labels from all packed items were kept for further examination. For samples collected from restaurants, we checked menus and asked the wait staff twice about the name of the marine species served to confirm each seafood type. In some instances, when the wait staff was not well informed, we requested information directly from the restaurant manager or chef. When possible, we targeted high priced menu-listings, which are more prone to be substituted by cheaper species. In most cases, we took pictures of the menu list and served dishes.

### DNA extraction, PCR amplification, and sequencing

#### DNA extraction

A small section of muscle was excised from the inner part of all collected samples, except for samples SF2, SF27, SF73, and SF74 from which a piece of dorsal fin was collected. Tissues were rinsed with distilled water and preserved in 99% ethanol at -20°C for further DNA analysis. Genomic DNA was isolated using the cetyl-trimethylammonium bromide (CTAB) precipitation method [35] for muscle tissues and the standard phenol-chloroform protocol [36] for fin tissue samples. To rehydrate the tissue and to remove contaminants, the processed samples were soaked in distilled water prior to DNA extraction.

#### Full barcoding PCR amplification

Aiming to identify the largest number of seafood species that came from a variety of retailers and processors including a wide range of taxonomic groups, 10 different primer sets were utilized including FB from the mitochondrial COI and 16S rRNA genes, and the control region, and MB from the COI and 12S rRNA genes (Table 1). PCR amplifications for the COI gene were carried out using Folmer primers LCO1490/HCO2198 [17] for fishes and invertebrates, FishF1/FishR1 [37] and “cocktail” [38] primer sets for fishes, and a degenerated version of Folmer primers COIF-ALT/COIR-ALT designed for Veneridae [39] was used for surf clams. For scallop samples, we used the Pectinidae family-specific primer set Pect16BC [18], targeting the 5’ end of the mitochondrial 16S rRNA gene. For marlin samples, we used the primer set A/G [40], flanking the complete mitochondrial control region. All primer sets used in this study are listed in Table 1.

**Table 1:** PCR primer sets used in the amplification of samples analyzed in this study.

Gene/ Region	Primer name	Direction	Sequence (5’-3’)	Ta (°C)	Target group	Source
COI	LCO1490	Forward	GGTCAACAAATCATAAAGATATTGG	45–54	Universal	[17]
	HCO2198	Reverse	TAAACTTCAGGGTGACCAAAAAATCA			
	FishF1	Forward	TCAACCAACCACAAAGACATTGGCAC	50–54	Fish	[37]
	FishR1	Reverse	TAGACTTCTGGGTGGCCAAAGAATCA			
	Fish_miniA_F_t	Forward	CACGACGTTGTAAAACGACACIAAICAIAAAGAYATYGGC	46	Fish (mini-barcoding)	[23]
	Fish_miniA_R_t	Reverse	**GGATAACAATTTCACACAGG**AARAAAATYATAACRAAIGCRTGIGC			
	Fish_miniD_F_t	Forward	**CACGACGTTGTAAAACGAC**GGIACIGGITGRACIGTITAYCCYCC	50	Fish (mini-barcoding)	[23]
	Fish_miniD_R_t	Reverse	**GGATAACAATTTCACACAGG**GTRATICCIGCIGCIAGIAC			
	Fish_miniE_F_t	Forward	**CACGACGTTGTAAAACGAC**ACYAAICAYAAAGAYATIGGCAC	46	Fish (mini-barcoding)	[23]
	Fish_miniE_R_t	Reverse	**GGATAACAATTTCACACAGG**CTTATRTTRTTTATICGIGGRAAIGC			
	VF2_t1	Forward	**TGTAAAACGACGGCCAGT**CAACCAACCACAAAGACATTGGCAC	50–52	Fish	[38]
	FishF2_t1	Forward	**TGTAAAACGACGGCCAGT**CGACTAATCATAAAGATATCGGCAC			
	FishR2_t1	Reverse	**CAGGAAACAGCTATGAC**ACTTCAGGGTGACCGAAGAATCAGAA			
	FR1d_t1	Reverse	**CAGGAAACAGCTATGAC**ACCTCAGGGTGTCCGAARAAYCARAA			
	COIF-ALT	Forward	ACAAATCAYAARGAYATYGG	46	Venus Clams	[39]
	COIR-ALT	Reverse	TTCAGGRTGNCCRAARAAYCA			
16S	Pect16BCF	Forward	CGTACCTTTTGCATCATGG	60	Scallops	[18]
	Pect16BCR	Reverse	GCGTAATCCGTCTTGACAGT			
12S	MiFish-U-F	Forward	**TCGTCGGCAGCGTCAGATGTGTATAAGAGACAGGT**GTCGGTAAAACTCGTGCCAGC	69.6–50 (TD)	Fish (mini-barcoding)	[21]
	MiFish-U-R	Reverse	**GTCTCGTGGGCTCGGAGATGTGTATAAGAGACAG**CATAGTGGGGTATCTAATCCCAGTTTG			
CONTROL REGION	A	Forward	TTCCACCTCTAACTCCCAAAGCTAG	54	Teleosts	[40]
	G	Reverse	CGTCGGATCCCATCTTCAGTGTTATGCTT			

Primer tails (adapters) are highlighted in bold when present. TD touchdown PCR

PCR reactions were performed in a TurboCycler Blue-Ray (BioTech, Taipei, Taiwan), a ProFlex PCR System (Applied Biosystems, Foster City, CA, USA) or a Veriti 96 Well thermal cycler (Applied Biosystems, Foster City, CA, USA) using Maximo *Taq* DNA Polymerase 2X-preMix (GeneOn GmbH, Nurnberg, Germany), HotStarTaq Plus Master Mix (QIAGEN, Hilden, Germany) or Maximo *Taq* DNA Polymerase (GeneOn GmbH, Nurnberg, Germany). Most PCR amplifications using either Folmer or FishF1/FishR1 primer sets were performed using the following protocol: 1–2 μl of genomic DNA, 10 μl of Maximo *Taq* DNA Polymerase 2X-preMix (GeneOn GmbH, Nurnberg, Germany), 0.5 μM each primer, in 20 μl of total volume. Thermocycling conditions were as follows: initial denaturation for 5 min at 95°C, followed by 35 cycles of denaturation for 30 s at 95°C, annealing for 40 s at 54°C (FishF1/FishR1) or at 45°C (Folmer), and extension for 1 min at 72°C, followed by a final extension for 10 min at 72°C. Master mix and PCR protocols using the other two abovementioned PCR kits are detailed in S1 Table. The PCR amplification using the “cocktail” primer set was performed with the HotStarTaq Plus Master Mix (QIAGEN, Hilden, Germany) using PCR conditions described previously [38] with slight modifications: 38 amplification cycles and annealing temperature from 50 to 52°C (see S1 Table). Primer sets COIF-ALT/COIR-ALT and A/G (control region) were amplified with HotStarTaq Plus Master Mix (QIAGEN, Hilden, Germany) and the amplification protocols are also detailed in S1 Table. The Pectinidae primer set Pect16BC was amplified following the amplification protocol described in Marín et al. [18]. All PCR products were electrophoresed in a 1.5% agarose gel and visualized under UV light.

#### Mini-barcoding PCR amplification

Samples that failed PCR amplification with COI “full barcoding” (hereafter FB) primer sets were amplified using three mini-barcoding (hereafter MB) primer sets, namely Mini_SH-A, Mini_SH-D, and Mini_SH-E [23]. PCR amplification reactions were the same as described above. PCR amplification conditions were as follows: initial denaturation for 5 min at 94°C, followed by 35 cycles of denaturation for 30 s at 94°C, annealing for 30 s at 46°C (Mini_SH-A and Mini_SH-E) or at 50°C (Mini_SH-D), and extension for 30 s at 72°C, followed by a final extension for 10 min at 72°C. In addition, a primer set namely MiFish-U [21] designed for fish metabarcoding environmental DNA (eDNA) that targets a small fragment (163–185 bp) of the hypervariable region of the 12S rRNA gene was used as an MB marker. PCR protocols for MB sets Mini_SH-A, Mini_SH-D, Mini_SH-E, and MiFish-U using HotStarTaq Plus Master Mix (QIAGEN, Hilden, Germany) are detailed in S1 Table.

#### Sequencing

Positive amplification products were sequenced in both directions at Macrogen Inc. sequencing facilities (Korea) on an ABI 3730xl Genetic Analyzer (Applied Biosystems, Foster City, CA) and at the Laboratory of Molecular Genetics of IMARPE (Peru) on an ABI 3500 (Applied Biosystems, Foster City, CA). Sequencing primers for all markers used in this study are indicated in S1 Table.

#### Data analyses

All DNA sequencing electropherograms were manually checked and edited by removing ambiguous base calling and adapter “tail” sequences (when necessary) using MEGA 7 software [41]. Complementary strand sequences were aligned so that a contiguous consensus was obtained for each sample. To avoid the inclusion of putative nuclear copies of COI gene sequences (NUMTs), sequences were manually checked for indels and premature stop codons. Identification of DNA sequences at species level was accomplished using both the Barcode of Life Data System (BOLD, http://www.boldsystems.org) selecting “species level barcode records” database and the Basic Local Alignment Search Tool (BLAST) on the National Center for Biotechnology Information (NCBI, http://www.blast.ncbi.nlm.nih.gov/Blast.cgi) identification engine (BLASTn, highly similar sequences “megablast”). Since records deposited only in the BOLD database have been validated for both the DNA sequence and specimen data, we used this repository as our final criteria for identifying seafood species. Only sequences with a similarity index ≥98% were considered a valid match [42]. In cases of ambiguous results obtained from NCBI and BOLD databases, further phylogenetic analysis including DNA sequences from both databases was performed using the Neighbor-Joining (NJ) method with Kimura 2-parameter model (K2P) [43] and 1000 bootstrap replicates using MEGA 7 software, and the Bayesian analysis inference (BI) using MrBayes 3.2.6 [44]. The level of substitution saturation for COI datasets was evaluated using DAMBE 6 [45]. We used jModelTest 2 [46] under the Akaike and Bayesian information criterion (AIC and BIC) to find the best-fit model of evolution. Two runs were performed simultaneously, each with four Markov chains. The analyses were run for one or five million generations with sampling every 100 generations. The first 25% of the sampled trees were discarded as burn-in. Obtained phylogenetic trees were drawn using FigTree 1.4.2 program (http://tree.bio.ed.ac.uk/software/figtree/). The species names obtained by barcoding analyses were then compared to the corresponding common/market names included in the “List of main species from artisanal fish landings during 2017” (hereafter “FISHLANDINGS-2017 list”), kindly provided by the Artisanal Fishery Office at IMARPE, and also compared with the fish common names presented in Chirichigno and Cornejo (2001) [4]. Acceptable English market names were searched within the FDA Seafood List accessible from https://www.accessdata.fda.gov/scripts/fdcc/index.cfm?set=seafoodlist. Accepted marine scientific names were checked in The World Register of Marine Species (WORMS, available at http://www.marinespecies.org) and FishBase (available at http://www.fishbase.de) databases. For batoid classification, we followed the nomenclature proposed in Last et al. [47]. Furthermore, we checked the conservation status for each genetically identified species at the International Union for Conservation of Nature (IUCN Red List of Threatened Species) publically available from http://www.iucnredlist.org.

## Results and discussion

### Molecular identification performance

**Fig 1:**
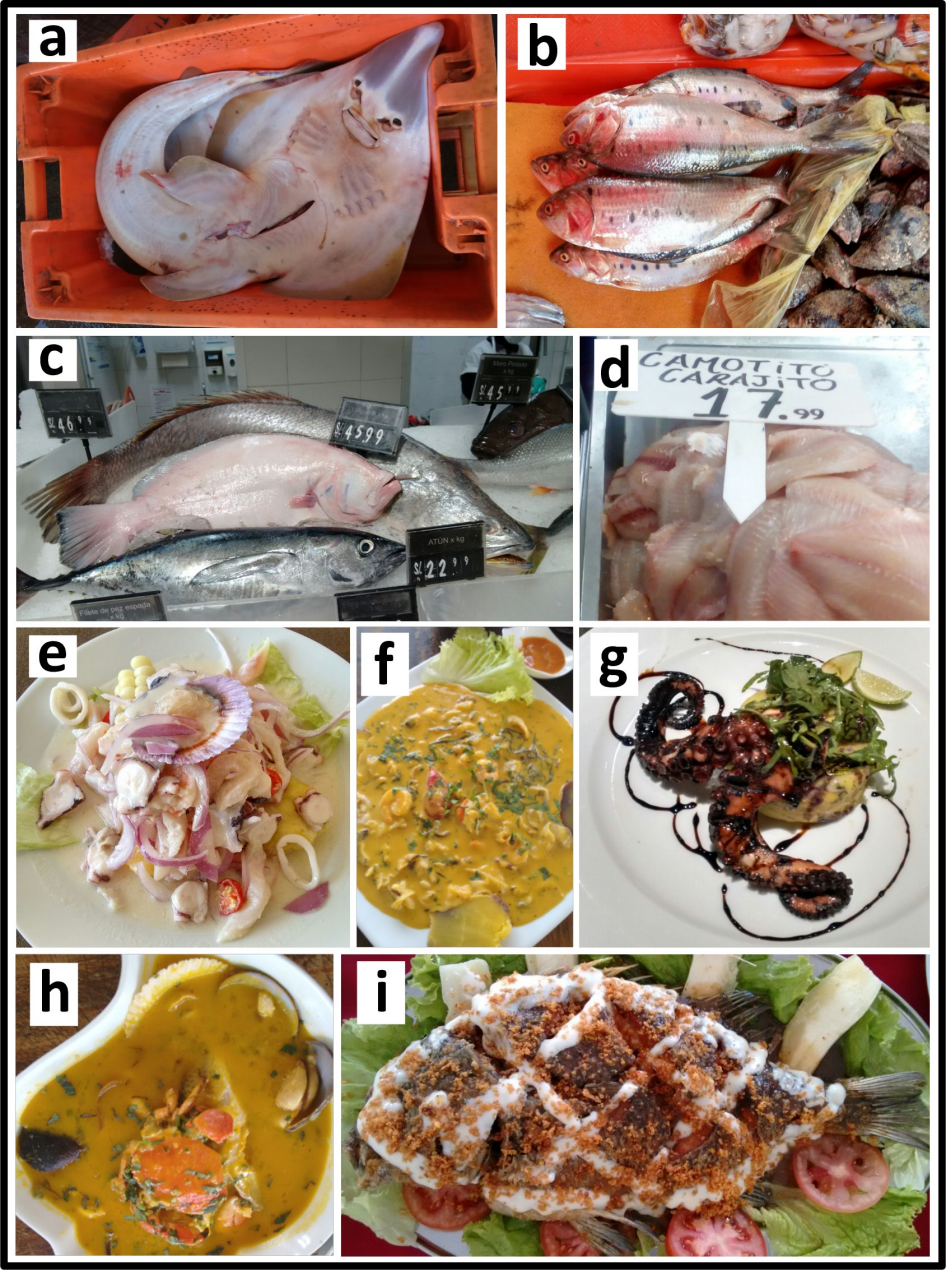
Representative pictures of sampling sites analyzed in this study. a. Fish Landing Site (FLS): guitarfish; b. Market (MK): Pacific menhaden; c. Supermarket chain (SMC); d. Multimarket (MM); e to i. Restaurant (RT): e. marinated seafood “*cebiche*”, f. spicy shellfish cream “*picante de mariscos*”, g. grilled octopus, h. fish and shellfish in “*parihuela*” soup, and i. fried Peruvian grunt.

This report represents the first intensive effort to accurately identify a wide range of commercial seafood products across the Peruvian supply chain (from harvest to consumption) using molecular markers. Overall, 137 PCR products were obtained, which represents a PCR success rate of 96%. Unsuccessful PCR amplifications resulted from canned and cooked samples, most likely due to DNA degradation or inhibitors presented in the processed food. We would like to emphasize that our protocols identified all “*cebiche*” samples (n = 16) collected from restaurants. Cebiche is the Peruvian national dish and by far the most popular and the pride of the citizens of Peru (see Fig 1E), where seafood is marinated with lime, which could affect the effectiveness of DNA isolation challenging downstream applications.

A total of 131 of the 137 amplified PCR products resulted in high quality sequencing electropherograms (sequencing success rate 96%), enabling proper seafood identification of 121 (92.37%) and 10 (7.63%) samples to species and genus level respectively. Of the 131 DNA sequences, 128 samples (97.71%) showed sequence identity greater than the threshold value (≥98%). However, because of either unresolved taxonomy or short variability of the COI gene among congeners, 13 samples (including octopus, marlin, smooth-hound, tilapia, and tuna specimens) matched with more than one species within 98% identity cutoff. Phylogenetic analyses for octopus, marlin, and smooth-hound samples are described below in subsections “Restaurants”, S1 Appendix, and S2 Appendix, respectively. Only three samples (SF42, SF44, and SF117) fell below the species-level identification cutoff (due to lack of reference sequences) and consequently, only a genus level identification was possible. Three (SF117, SF119, and SF128) of the 115 specimens identified with FB (COI) did not match to any records in the BOLD database.

### Mini-barcoding efficiency

The efficiency of all the MB primers tested in this study is presented in S2 Table. Species identities within the range of 98–100% for NCBI and 98.2–100% for BOLD databases were obtained in 15 tested fish samples, of which 14 could not be amplified with FB markers. Most of those included canned and cooked samples from SMCs and RTs such as fish fritters, tortilla, fried, and steamed. A canned tuna sample (SF68) was successfully amplified and sequenced by MB, but BLAST analysis showed multiple *Thunnus* species (*T*. *atlanticus*, *T*. *orientalis*, and *T*. *albacares*) matching at 98% identity. A low genetic distance among those tuna species hampers a species level identification through DNA barcoding, consequently multilocus approaches based on control region with ITS1 [48] and 12S with ND5 markers [21] have been recommended.

Among the three tested MB primer sets from Shokralla et al. [23], the primer sets SH-A and SH-E showed the highest PCR amplification (63% and 77% respectively) and sequencing success rates (100% and 90% respectively). All three primer sets (SH-A, SH-D, and SH-E) showed high sequence identity (above 98%, S2 Table), which allowed a correct identification of eight bony fishes, one shark, and one devil ray species. A shark filet (SF76) was identified only to the genus level due to lack of reference sequence. Interestingly, this study added elasmobranch species to the performance test of these MB primers with promising results, however, we tested only a limited number of shark and devil ray species. Further analyses using a wider range of elasmobranch species are needed to find out the efficacy of those MB primers in the identification of members of this fish group. Mini-barcoding primer sets SH-A, SH-D, and SH-E failed to amplify one canned herring sample SF77 (Marine Stewardship Council -MSC- certified). Therefore, we tested an additional primer set MiFish-U targeting a small fragment of the 12S rRNA gene [21]. This primer set showed a high performance by identifying sample SF77 as *Clupea harengus* with 100% identity (NCBI database), confirming its correct label information. Using the same primer set, a second canned sample (SF96) was also tested and identified as *Sardina pilchardus* with 100% identity (NCBI database). Sample SF96 was also identified with primer sets SH-A and SH-E with sequence similarities of 99.22% and 99.56%, respectively, in the BOLD database. Our results indicate that the primer set MiFish-U designed by Miya et al. [21] can be considered an alternative potential MB marker for the identification of canned fish samples. Overall, the MB approach applied in this study revealed four cases of misbranding, which are shown in S3 Table.

### Species diversity

Even though our sampling was performed in a relatively small number of retailers, which included 21 RTs, eight MKs, seven stores from four SMCs, six FLSs, one WFM, one GS, and one MM, a highly diversified fauna including both marine (93.44%) and freshwater (6.56%) species was found over the 20-month sampling period. We identified 55 species (plus five genus-level taxa) covering 47 families, 24 orders, and six classes including Actinopterygii (45.03%), Chondrichthyes (36.64%), Bivalvia (6.87%), Cephalopoda (6.11%), Malacostraca (3.82%), and Gastropoda (1.53%) (Table 2). The most diverse group was Perciformes represented by 12 families, 16 genera, and 16 species, followed by Myliobatiformes with five families, six genera, and six species. All seafood DNA sequences obtained in this study have been deposited in GenBank/EMBL/DDBJ databases with accession numbers from MH194422 to MH194552 and from MK070511 to MK070524 (Table 3).

**Table 2:** Taxonomic classification of seafood diversity identified in this study. Conservation status was retrieved from IUCN Red List. IUCN abbreviations: NE Not Evaluated, DD Data Deficient, LC Least Concern, NT Near Threatened, VU Vulnerable, EN Endangered. n: sample size. Identification to the genus level (ID% or Similarity% <98) is highlighted in bold.

Seafood type	Class	Order	Family	Scientific name	Common name English/Spanish	IUCN	n	Retailer (sample code)
**Fish**	**Actinopterygii **	Atheriniformes	Atherinopsidae	*Odontesthes regia* (Humboldt, 1821)	Peruvian Silverside/Pejerrey	LC	2	RT-LI (SF97, SF129)
		Clupeiformes	Clupeidae	Clupea harengus* Linnaeus, 1758	Atlantic Herring/Arenque	LC	1	SMC-LI (SF77)
				*Ethmidium maculatum* (Valenciennes, 1847)	Pacific Menhaden/Machete	DD	2	MK-LI (SF73, SF74)
				Sardina pilchardus* (Walbaum, 1792)	European Pilchard/Sardina Europea	LC	2	SMC-LI (SF93, SF96)
			Engraulidae	*Engraulis ringens* Jenyns, 1842	Anchovy/Anchoveta	LC	4	SMC-LL (SF69, SF70) SMC-AN (SF116) MM-LI (SF92)
		Gadiformes	Merluccidae	*Merluccius gayi* (Guichenot, 1848)	South Pacific Hake/Merluza	DD	1	RT-LL (SF7)
		Lampriformes	Lampridae	*Lampris guttatus* (Brünnich, 1788)	Opah, Moonfish/Pez Luna	LC	1	MM-LI (SF84)
		Perciformes	Carangidae	*Seriola rivoliana* Valenciennes, 1833	Almaco Jack/Fortuno	LC	1	RT-LI (SF125)
			Centrolophidae	*Schedophilus haedrichi* Chirichigno F., 1973	Mocosa Ruff/Cojinoba Mocosa, Ojo de Uva	LC	2	MK-LL (SF2) RT-LL (SF25)
			Cichlidae	***Oreochromis* sp.**	Tilapia/Tilapia	-	2	MM-LI (SF88, SF89)
			Coryphaenidae	*Coryphaena hippurus* Linnaeus, 1758	Dolphinfish/Perico	LC	10	FLS-TU (SF28) SMC-AN (SF114) SMC-LI (SF91) MM-LI (SF90) RT-LL (SF17, SF23) RT-LI (SF99, SF100, SF121, SF130)
			Haemulidae	*Anisotremus interruptus* (Gill, 1862)	Burrito Grunt/Sargo, Chita Dorada	LC	1	MK-LL (SF27)
				*Anisotremus scapularis* (Tschudi, 1846)	Peruvian Grunt/Chita	LC	4	MK-AN (SF105) RT-LL (SF22), RT-PI (SF79) RT-AN (SF101)
			Istiophoridae	Kajikia albida* (Poey, 1860)	Atlantic White Marlin/Merlín Blanco	**VU**	1	MM-LI (SF131)
				*Kajikia audax* (Philippi, 1887)	Striped Marlin/Merlín Rayado	NT	1	FLS-TU (SF65)
			Labrisomidae	*Labrisomus philippii* (Steindachner, 1866)	Chalapo Clinid/Trambollo	LC	1	RT-LI (SF98)
			Sciaenidae	*Cheilotrema fasciatum* Tschudi, 1846	Arnillo Drum/Burro	NE	1	MK-AN (SF106)
				*Cynoscion praedatorius* (Jordan & Gilbert, 1889)	Boccone Weakfish/Corvina Bocona	DD	1	RT-LL (SF26)
				*Paralonchurus peruanus* (Steindachner, 1875)	Peruvian Croaker/Suco	LC	1	RT-LL (SF21)
			Scombridae	*Sarda chiliensis* (Cuvier, 1832)	Pacific Bonito/Bonito	LC	1	RT-LI (SF94)
				***Thunnus* sp.**	Tuna/Atún	-	1	GS-LL (SF68)
			Serranidae	*Mycteroperca xenarcha* Jordan, 1888	Broomtail Grouper/Mero Negro	LC	1	RT-LI (SF122)
				*Paralabrax humeralis* (Valenciennes, 1828)	Peruvian Rock Seabass/Cabrilla	DD	3	RT-AN (SF103, SF120) RT-PI (SF78)
			Sphyraenidae	*Sphyraena ensis* Jordan & Gilbert, 1882	Barracuda/Barracuda, Picuda	LC	1	MM-LI (SF126)
			Xiphiidae	*Xiphias gladius* Linnaeus, 1758	Swordfish/Pez Espada	LC	4	FLS-TU (SF45) MK-LL (SF11) RT-LL (SF8, SF9)
		Pleuronectiformes	Paralichthyidae	*Etropus ectenes* Jordan, 1889	Sole Flounder/Lenguado	LC	2	RT-LL (SF3, SF4)
		Salmoniformes	Salmonidae	*Oncorhynchus mykiss* (Walbaum, 1792)	Rainbow Trout/Trucha Arco Iris	NE	1	SMC-AN (SF108)
				Salmo salar* Linnaeus, 1758	Atlantic Salmon/Salmón	LC	1	MM-LI (SF95)
		Scorpaeniformes	Triglidae	*Prionotus stephanophrys* Lockington, 1881	Lumpfish Searobin/Cabrilla Voladora	LC	2	MK-AN (SF110) RT-AN (SF71)
		Siluriformes	Pangasiidae	Pangasianodon hypophthalmus* (Sauvage, 1878)	Striped Pangasius, Swai/Basa	**EN**	3	SMC-AN (SF111, SF113) SMC-LI (SF87)
**Sharks**	**Chondrichthyes**	Carcharhiniformes	Carcharhinidae	*Prionace glauca* (Linnaeus, 1758)	Blue Shark/Tiburón Azul	NT	5	MK-LA (SF63) SMC-LL (SF59, SF60) RT-LL (SF19, SF20)
			Sphyrnidae	*Sphyrna zygaena* (Linnaeus, 1758)	Smooth Hammerhead/Tiburón Martillo	**VU**	9	FLS-TU (SF66, SF83) WFM-LL (SF50-SF54) MK-LL (SF10) RT-LL (SF1)
			Triakidae	*Mustelus lunulatus* Jordan & Gilbert, 1882	Sicklefin Smooth-hound/Tollo	LC	1	SMC-LI (SF75)
				***Mustelus* sp.**	Smooth-hound/Tollo	-	4	WFM-LL (SF56) SMC-LL (SF57) SMC-LI (SF76) MM-LI (SF85)
		Lamniformes	Alopiidae	*Alopias pelagicus* Nakamura, 1935	Pelagic Thresher Shark/Tiburón Zorro Pelágico	**VU**	3	WFM-LL (SF55) MK-TU (SF35) MK-LL (SF12)
			Lamnidae	*Isurus oxyrinchus* Rafinesque, 1810	Shortfin Mako Shark/Tiburón Diamante	**VU**	1	MM-LI (SF86)
		Orectolobiformes	Rhincodontidae	*Rhincodon typus* Smith, 1828	Whale Shark/Tiburón Ballena	**EN**	1	FLS-TU (SF46)
**Rays and their relatives**		Myliobatiformes	Aetobatidae	*Aetobatus laticeps* Gill, 1865	Pacific Spotted Eagle Ray/Raya Pintada	NE	1	FLS-TU (SF49)
			Dasyatidae	*Hypanus dipterurus* (Jordan & Gilbert, 1880)	Diamond Stingray/Raya Látigo, Batana, Batea	DD	1	FLS-TU (SF33)
				*Pteroplatytrygon violacea* (Bonaparte, 1832)	Pelagic Stingray/Raya Negra, Raya Látigo Coluda	LC	1	FLS-TU (SF37)
			Gymnuridae	***Gymnura* sp.**	Butterfly Ray/Raya Mariposa	-	2	FLS-TU (SF42, SF44)
			Mobulidae	*Mobula mobular* (Bonnaterre, 1788)	Giant Devil Ray/Manta	EN**	10	FLS-TU (SF30, SF32, SF34, SF36, SF48) MK-TU (SF31, SF40, SF43) MK-LA (SF61) RT-LL (SF18)
			Myliobatidae	*Myliobatis chilensis* Philippi, 1892	Chilean Eagle Ray/Raya Águila	DD	4	MK-LA (SF62, SF64) MK-LL (SF29) SMC-LL (SF58)
				*Myliobatis longirostris* Applegate & Fitch, 1964	Longnose Eagle Ray/Raya Picuda	NT	1	FLS-TU (SF41)
		Rajiformes	Arhynchobatidae	*Sympterygia brevicaudata* (Cope, 1877)	Shorttail Fanskate/Pastelillo	DD	1	FLS-TU (SF47)
		Rhinopristiformes	Rhinobatidae	*Pseudobatos planiceps* (Garman, 1880)	Pacific Guitarfish/Pez Guitarra	DD	1	FLS-TU (SF39)
		Torpediniformes	Narcinidae	*Narcine entemedor* Jordan & Starks, 1895	Giant Electric Ray/Raya Eléctrica	DD	2	FLS-TU (SF38, SF67)
**Shellfish**								
**Crustaceans**	**Malacostraca**	Decapoda	Cancridae	*Romaleon setosum* (Molina, 1782)	Hairy Crab/Cangrejo Peludo	NE	1	SMC-AN (SF115)
			Penaeidae	Metapenaeus dobsoni* (Miers, 1878)	Kadal Shrimp/Langostino	NE	1	SMC-LL (SF82)
				*Penaeus vannamei* Boone, 1931	Whiteleg Shrimp/Langostino	NE	2	SMC-AN (SF107) RT-LL (SF16)
			Platyxanthidae	*Platyxanthus orbignyi* (H. Milne Edwards & Lucas, 1843)	Crab/Cangrejo Violáceo	NE	1	RT-LL (SF24)
**Mollusks**	**Bivalvia**	Arcida	Arcidae	*Anadara tuberculosa* (G.B. Sowerby I, 1833)	Mangrove Cockle/Concha Negra	NE	1	RT-LL (SF15)
		Cardiida	Donacidae	*Donax obesulus* Reeve, 1854	Surf Clam/Maruchita, Palabrita	NE	2	MK-AN (SF119) MM-LI (SF128)
			Semelidae	***Semele* sp.**	Clam/Almeja	-	1	MK-AN (SF117)
			Solecurtidae	*Tagelus dombeii* (Lamarck, 1818)	Jackknife Clam/Navajuela	NE	3	MK-AN (SF118) MM-LI (SF127) RT-AN (SF102)
		Pectinida	Pectinidae	*Argopecten purpuratus* (Lamarck, 1819)	Peruvian Scallop/Concha de Abanico	NE	2	RT-LL (SF13) RT-LI (SF123)
	**Cephalopoda**	Myopsida	Loliginidae	*Doryteuthis (Amerigo) gahi* (d’Orbigny, 1835)	Patagonian Squid/Calamar Común	NE	1	RT-PI (SF80)
		Octopoda	Octopodidae	*Octopus mimus* Gould, 1852	Gould Octopus/Pulpo Común	NE	4	RT-LL (SF14) RT-AN (SF81) RT-LI (SF72, SF124)
		Oegopsida	Ommastrephidae	*Dosidicus gigas* (d’Orbigny [in 1834–1847], 1835)	Humboldt Squid/Pota, Calamar Gigante	DD	3	SMC-AN (SF112) RT-LL (SF5, SF6)
	**Gastropoda**	Neogastropoda	Muricidae	*Thaisella chocolata* (Duclos, 1832)	Sea Snail/Caracol	NE	2	MK-AN (SF109) RT-AN (SF104)
**Total**	6	24	47	55 species			131	

Retailer categories: FLS Fish Landing Site, WFM Wholesale Fish Market, MK Market, GS Grocery Store, SMC SuperMarket Chain, MM MultiMarket, RT Restaurant

Locations: TU Tumbes, PI Piura, LA Lambayeque, LL La Libertad, AN Ancash, and LI Lima

* Species detected in imported products

** Awaiting reassessment due to synonymization with *M*. *japanica*

**Table 3:** Species identification results of the 131 samples collected through the supply chain of the Peruvian fishery sector using full (FB) and mini-barcoding (MB), results are based in NCBI and BOLD databases.

Code	Sampling date	Labeled or declared as (English/Spanish)	Presentation	Retailer	Location	NCBI		BOLD		Common Name English/Spanish	Primer/ Marker (Full-Mini barcode)	GenBank accession number	Misla-beled
						Species name	ID (%)	Species name (Accepted)	Simila-rity (%)				
SF1	2016.07.20	Smooth-hound/Tollo	Fish fritters	Restaurant	Trujillo (LL)	*Sphyrna zygaena*	99	*Sphyrna zygaena*	100	Smooth Hammerhead/Tiburón Martillo	FISH1/COI (FB)	MH194422	**YES**
SF2	2016.07.21	Mocosa Ruff/Ojo de Uva	Whole body	Market	Trujillo (LL)	*Icichthys lockingtoni*	96	*Schedophilus haedrichi*	99.44	Mocosa Ruff/Cojinoba Mocosa, Ojo de Uva	SH-E/COI (MB)	MH194423	NO
SF3	2016.07.22	Flounder/Lenguado	Marinated “cebiche”	Restaurant	Trujillo (LL)	*Etropus crossotus*	88	*Etropus ectenes*	100	Sole Flounder/Lengueta, Lenguado	Folm/COI (FB)	MH194424	NO
SF4	2016.07.22	Flounder/Lenguado	Marinated “cebiche”	Restaurant	Trujillo (LL)	*Etropus crossotus*	88	*Etropus ectenes*	100	Sole Flounder/Lengueta, Lenguado	Folm/COI (FB)	MH194425	NO
SF5	2016.07.22	Humboldt Squid/Pota	Marinated “cebiche”	Restaurant	Trujillo (LL)	*Dosidicus gigas*	100	*Dosidicus gigas*	100	Humboldt Squid/Calamar Gigante, Pota	Folm/COI (FB)	MH194426	NO
SF6	2016.07.22	Humboldt Squid/Pota	Marinated “cebiche”	Restaurant	Trujillo (LL)	*Dosidicus gigas*	100	*Dosidicus gigas*	100	Humboldt Squid/Calamar Gigante, Pota	Folm/COI (FB)	MH194427	NO
SF7	2016.07.24	Grouper/Mero	Marinated “cebiche”	Restaurant	Huanchaco (LL)	*Merluccius gayi*	100	*Merluccius gayi*	100	South Pacific Hake/Merluza	FISH1/COI (FB)	MH194428	**YES**
SF8	2016.07.24	Grape-eye Seabass/Ojo de Uva	Fish fritters	Restaurant	Huanchaco (LL)	*Xiphias gladius*	100	*Xiphias gladius*	100	Swordfish/Pez Espada	SH-D/COI (MB)	MH194429	**YES**
SF9	2016.07.24	Grape-eye Seabass/Ojo de Uva	Fish fritters	Restaurant	Huanchaco (LL)	*Xiphias gladius* *Xiphias gladius*	100 100	*Xiphias gladius* *Xiphias gladius*	100 100	Swordfish/Pez Espada	SH-E/COI (MB) SH-A/COI (MB)	MH194430 MK070512	**YES**
SF10	2016.07.25	Thresher Shark/Tollo Zorro	Fresh, headless, no caudal fin	Market	Trujillo (LL)	*Sphyrna zygaena* *Sphyrna zygaena*	100 100	*Sphyrna zygaena* *Sphyrna zygaena*	100 100	Smooth Hammerhead/Tiburón Martillo	SH-E/COI (MB) SH-A/COI (MB)	MH194431 MK070513	**YES**
SF11	2016.07.25	Swordfish/Pez Espada	Fresh, headless, finless	Market	Trujillo (LL)	*Xiphias gladius*	100	*Xiphias gladius*	100	Swordfish/Pez Espada	FISH1/COI (FB)	MH194432	NO
SF12	2016.07.25	Smooth-hound/Tollo	Fresh, headless, finless	Market	Trujillo (LL)	*Alopias pelagicus*	100	*Alopias pelagicus*	100	Pelagic Thresher Shark/Tiburón Zorro Pelágico	FISH1/COI (FB)	MH194433	**YES**
SF13	2016.07.27	Peruvian Scallop/Concha de Abanico	Marinated “cebiche”	Restaurant	Trujillo (LL)	*Argopecten purpuratus*	99	NO MATCH	-	Peruvian Scallop/Concha de Abanico	Pect16/16S (FB)	MH194434	NO
SF14	2016.07.27	Octopus/Pulpo	Marinated “cebiche”	Restaurant	Trujillo (LL)	*Octopus mimus*, *O*. *hubbsorum*	100	*Octopus hubbsorum*	100	Hubb’s Octopus/Pulpo Verde	Folm/COI (FB)	MH194435	NO
SF15	2016.07.27	Mangrove Cockle/Concha Negra	Marinated “cebiche”	Restaurant	Trujillo (LL)	*Anadara tuberculosa*	99	*Anadara tuberculosa*	98	Mangrove Cockle/Concha Negra	Folm/COI (FB)	MH194436	NO
SF16	2016.07.27	Shrimp/Langostino	Marinated “cebiche”	Restaurant	Trujillo (LL)	*Litopenaeus vannamei*	100	*Litopenaeus vannamei* **(*Penaeus vannamei)***	100	Whiteleg Shrimp/Langostino	Folm/COI (FB)	MH194437	NO
SF17	2016.08.02	Grouper/Mero	Steamed, filet “sudado”	Restaurant	Huanchaco (LL)	*Coryphaena hippurus*	99	*Coryphaena hippurus*	100	Dolphinfish/Perico, Dorado	FISH1/COI (FB)	MH194438	**YES**
SF18	2016.08.03	Ray/Raya	Tortilla	Restaurant	Trujillo (LL)	*Mobula mobular*, *M*. *japanica* *M*. *japanica*, *M*. *mobular*	100 100	*Mobula japanica* **(*M*. *mobular*)** *Mobula japanica* **(*M*. *mobular*)**	100 100	Spinetail Devil Ray/Manta	SH-E/COI (MB) SH-A/COI (MB)	MH194439 MK070514	**YES**
SF19	2016.08.05	Smooth-hound/Tollo	Fish fritters	Restaurant	Trujillo (LL)	*Prionace glauca*	100	*Prionace glauca*	100	Blue Shark/Tiburón Azul	Folm/COI (FB)	MH194440	**YES**
SF20	2016.08.05	Smooth-hound/Tollo	Fish fritters	Restaurant	Trujillo (LL)	*Prionace glauca*	100	*Prionace glauca*	100	Blue Shark/Tiburón Azul	Folm/COI (FB)	MH194441	**YES**
SF21	2016.08.10	Croaker/Suco	Steamed, whole body	Restaurant	Trujillo (LL)	*Paralonchurus peruanus*	100	*Paralonchurus peruanus*	100	Peruvian Croaker/Suco	SH-D/COI (MB)	MH194442	NO
SF22	2016.08.11	Peruvian Grunt/Chita	Fried, whole body, with garlic sauce	Restaurant	Moche (LL)	*Anisotremus scapularis*	100	*Anisotremus scapularis*	100	Peruvian Grunt/Chita	FISH1/COI (FB)	MH194443	NO
SF23	2016.08.11	Corvina/Corvina	Marinated “cebiche”	Restaurant	Moche (LL)	*Coryphaena hippurus*	99	*Coryphaena hippurus*	100	Dolphinfish/Perico, Dorado	Folm/COI (FB)	MH194444	**YES**
SF24	2016.08.14	Crab/Cangrejo	Marinated “cebiche”	Restaurant	Salaverry (LL)	*Platyxanthus orbignyi*	100	*Platyxanthus orbignyi*	100	Purple Crab/Cangrejo Violáceo	Folm/COI (FB)	MH194445	NO
SF25	2016.08.19	Corvina/Corvina	Steamed, filet “sudado”	Restaurant	Huanchaco (LL)	*Schedophilus labyrinthicus*	94	*Schedophilus haedrichi*	100	Mocosa Ruff/Cojinoba Mocosa	FISH1/COI (FB)	MH194446	**YES**
SF26	2016.08.21	Corvina/Corvina	Fried	Restaurant	Trujillo (LL)	*Cynoscion praedatorius*	100	*Cynoscion praedatorius*	100	Boccone Weakfish/Corvina Bocona	FISH1/COI (FB)	MH194447	NO
SF27	2016.09.07	Burrito Grunt/Sargo Dorado	Fresh, whole body	Market	Trujillo (LL)	*Anisotremus interruptus* *Anisotremus interruptus*	99 99	*Anisotremus interruptus* *Anisotremus interruptus*	100 100	Burrito Grunt/Chita Dorada, Sargo Dorado	SH-E/COI (MB) SH-A/COI (MB)	MH194448 MK070515	NO
SF28	2016.11.13	Dolphinfish/Perico	Fresh, whole body	Fish landing site	La Cruz (TU)	*Coryphaena hippurus*	100	*Coryphaena hippurus*	100	Dolphinfish/Perico, Dorado	FISH1/COI (FB)	MH194449	NO
SF29	2016.12.08	Manta/Manta	Dried cured	Market	Trujillo (LL)	*Myliobatis chilensis*	99	*Myliobatis chilensis*	99.85	Chilean Eagle Ray/Raya Águila	Folm/COI (FB)	MH194450	**YES**
SF30	2017.01.23	Black Manta/Mantarraya Negra	Fresh, whole body	Fish landing site	La Cruz (TU)	*Mobula japanica*, *M*. *mobular*	100	*Mobula japanica* **(*M*. *mobular*)**	100	Spinetail Devil Ray/Manta	FISH1/COI (FB)	MH194451	NO
SF31	2017.01.23	Manta Ray/Mantarraya	Fresh filet	Market	Tumbes (TU)	*Mobula japanica*, *M*. *mobular*	100	*Mobula japanica* **(*M*. *mobular*)**	100	Spinetail Devil Ray/Manta	FISH1/COI (FB)	MH194452	NO
SF32	2017.01.23	Manta Ray/Mantarraya	Fresh, whole body	Fish landing site	La Cruz (TU)	*Mobula japanica* *M*. *mobular* *M*. *japanica*, *M*. *mobular*	100 99	*Mobula japanica* **(*M*. *mobular*)** *Mobula japanica* **(*M*. *mobular*)**	100 100	Spinetail Devil Ray/Manta	FISH1/COI (FB) Folm/COI (FB)	MH194453 MK070516	NO
SF33	2017.01.24	Batea Ray/Batea	Fresh, whole body	Fish landing site	Acapulco (TU)	*Dasyatis brevis*	96	*Dasyatis dipterura* **(*Hypanus dipterurus)***	100	Diamond Stingray/Batana, Batea, Raya Látigo	FISH1/COI (FB)	MH194454	NO
SF34	2017.01.24	Manta/Manta	Fresh, whole body	Fish landing site	Zorritos (TU)	*Mobula japanica*, *M*. *mobular*	100	*Mobula japanica* **(*M*. *mobular*)**	100	Spinetail Devil Ray/Manta	FISH1/COI (FB)	MH194455	NO
SF35	2017.01.24	Shortfin Mako/Tollo Diamante	Fresh filet	Market	Zorritos (TU)	*Alopias pelagicus*	100	*Alopias pelagicus*	100	Pelagic Thresher Shark/Tiburón Zorro Pelágico	FISH1/COI (FB)	MH194456	**YES**
SF36	2017.01.24	Manta/Manta	Fresh fins	Fish landing site	Cancas (TU)	*Mobula japanica*, *M*. *mobular*	100	*Mobula japanica* **(*M*. *mobular*)**	100	Spinetail Devil Ray/Manta	FISH1/COI (FB)	MH194457	NO
SF37	2017.01.24	Black Stingray/Raya Coluda Negra	Fresh, whole body	Fish landing site	Punta Mero (TU)	*Pteroplatytrygon violacea*	99	*Pteroplatytrygon violacea*	100	Pelagic Stingray/Raya Negra, Raya Látigo Coluda	Folm/COI (FB)	MH194458	NO
SF38	2017.01.24	Electric Ray/Raya Eléctrica	Fresh, whole body	Fish landing site	Acapulco (TU)	*Narcine entemedor* *Narcine entemedor*	99 99	*Narcine entemedor* *Narcine entemedor*	100 100	Giant Electric Ray/Raya Eléctrica	FISH1/COI (FB) Folm/COI (FB)	MH194459 MK070517	NO
SF39	2017.01.24	Guitarfish/Pez Guitarra	Fresh, whole body	Fish landing site	Acapulco (TU)	*Rhinobatos glaucostigma*	97	*Pseudobatos planiceps*	100	Pacific Guitarfish/Pez Guitarra	Folm/COI (FB)	MH194460	NO
SF40	2017.01.24	Manta Ray/Mantarraya	Fresh filet	Market	Zorritos (TU)	*Mobula japanica*, *M*. *mobular*	100	*Mobula japanica* **(*M*. *mobular*)**	100	Spinetail Devil Ray/Manta	FISH1/COI (FB)	MH194461	NO
SF41	2017.01.24	Stingray/Raya Coluda	Fresh, whole body	Fish landing site	Punta Mero (TU)	*Aetobatus narinari*	95	*Myliobatis longirostris*	98.74	Longnose Eagle Ray/Raya Picuda	FISH1/COI (FB)	MH194462	NO
SF42	2017.01.24	Butterfly Ray/Tuyo	Fresh filet	Fish landing site	Acapulco (TU)	*Gymnura micrura*	87	*Gymnura *marmorata*	97.65	California Butterfly Ray/Raya Tuyo, Raya Mariposa	FISH1/COI (FB)	MH194463	NO
SF43	2017.01.24	Manta/Manta	Fresh filet	Market	Zorritos (TU)	*Mobula japanica*, *M*. *mobular*	100	*Mobula japanica* **(*M*. *mobular*)**	100	Spinetail Devil Ray/Manta	FISH1/COI (FB)	MH194464	NO
SF44	2017.01.24	Butterfly Ray/Raya Tuyo, Raya Mariposa	Fresh, whole body	Fish landing site	Punta Sal (TU)	*Gymnura micrura*	87	*Gymnura *marmorata*	97.7	California Butterfly Ray/Raya Tuyo, Raya Mariposa	FISH1/COI (FB)	MH194465	NO
SF45	2017.01.24	Swordfish/Pez Espada	Fresh, headless	Fish landing site	Zorritos (TU)	*Xiphias gladius*	99	*Xiphias gladius*	100	Swordfish/Pez Espada	FISH1/COI (FB)	MH194466	NO
SF46	2017.01.24	Whale Shark/Tiburón Ballena	Fresh, whole body	Fish landing site	Acapulco (TU)	*Rhincodon typus*	100	*Rhincodon typus*	100	Whale Shark/Tiburón Ballena	Folm/COI (FB)	MH194467	NO
SF47	2017.01.25	Witch Skate/Raya Bruja	Fresh, whole body	Fish landing site	Cancas (TU)	*Sympterygia brevicaudata*	99	*Sympterygia brevicaudata*	100	Shorttail Fanskate/Pastelillo	Folm/COI (FB)	MH194468	**YES**
SF48	2017.01.25	Manta/Manta	Fresh, fins	Fish landing site	Cancas (TU)	*Mobula japanica*, *M*. *mobular*	100	*Mobula japanica* **(*M*. *mobular*)**	100	Spinetail Devil Ray/Manta	FISH1/COI (FB)	MH194469	NO
SF49	2017.01.25	Spotted Ray/Raya Pintada	Fresh, whole body	Fish landing site	Cancas (TU)	*Aetobatus narinari*	99	*Aetobatus narinari* **(*Aetobatus laticeps*)**	100	Pacific Spotted Eagle Ray/Raya Pintada	FISH1/COI (FB)	MH194470	NO
SF50	2017.01.28	Thresher Shark/Tollo Zorro	Fresh, headless	Wholesale fish market	Buenos Aires (LL)	*Sphyrna zygaena*	99	*Sphyrna zygaena*	100	Smooth Hammerhead/Tiburón Martillo	FISH1/COI (FB)	MH194471	**YES**
SF51	2017.01.28	Thresher Shark/Tollo Zorro	Fresh, headless	Wholesale fish market	Buenos Aires (LL)	*Sphyrna zygaena*	100	*Sphyrna zygaena*	100	Smooth Hammerhead/Tiburón Martillo	FISH1/COI (FB)	MH194472	**YES**
SF52	2017.01.28	Thresher Shark/Tollo Zorro	Fresh, headless	Wholesale fish market	Buenos Aires (LL)	*Sphyrna zygaena*	100	*Sphyrna zygaena*	100	Smooth Hammerhead/Tiburón Martillo	FISH1/COI (FB)	MH194473	**YES**
SF53	2017.01.28	Thresher Shark/Tollo Zorro	Fresh, headless	Wholesale fish market	Buenos Aires (LL)	*Sphyrna zygaena*	100	*Sphyrna zygaena*	100	Smooth Hammerhead/Tiburón Martillo	FISH1/COI (FB)	MH194474	**YES**
SF54	2017.01.28	Thresher Shark/Tollo Zorro	Fresh, headless	Wholesale fish market	Buenos Aires (LL)	*Sphyrna zygaena*	100	*Sphyrna zygaena*	100	Smooth Hammerhead/Tiburón Martillo	FISH1/COI (FB)	MH194475	**YES**
SF55	2017.01.28	Thresher Shark/Tollo Zorro	Fresh, headless	Wholesale fish market	Buenos Aires (LL)	*Alopias pelagicus*	99	*Alopias pelagicus*	100	Pelagic Thresher Shark/Tiburón Zorro Pelágico	Folm/COI (FB)	MH194476	NO
SF56	2017.01.28	Smooth-hound/Tollo Mamita	Fresh filet	Wholesale fish market	Buenos Aires (LL)	*Mustelus henlei*	98	**Mustelus *henlei*	98.55	Brown Smooth-hound/Tollo	FISH1/COI (FB)	MH194477	NO
SF57	2017.01.31	Smooth-hound/Tollo	Refrigerated filet	Supermarket chain	Trujillo (LL)	*Mustelus henlei*, *M*. *canis*	98	**Mustelus *henlei*	98.45	Brown Smooth-hound/Tollo	FISH1/COI (FB)	MH194478	NO
SF58	2017.01.31	Guitarfish/Pez Guitarra	Refrigerated filet	Supermarket chain	Trujillo (LL)	*Myliobatis chilensis*	100	*Myliobatis chilensis*	100	Chilean Eagle Ray/Raya Águila	FISH1/COI (FB)	MH194479	**YES**
SF59	2017.01.31	Blue Shark/Tollo Azul	Frozen filet	Supermarket chain	Trujillo (LL)	*Prionace glauca*	99	*Prionace glauca*	100	Blue Shark/Tiburón Azul	Folm/COI (FB)	MH194480	NO
SF60	2017.01.31	Shark/Tiburón	Refrigerated filet	Supermarket chain	Trujillo (LL)	*Prionace glauca*	100	*Prionace glauca*	100	Blue Shark/Tiburón Azul	Folm/COI (FB)	MH194481	NO
SF61	2017.02.01	Manta/Manta	Dried	Market	Chiclayo (LA)	*Mobula japanica*, *M*. *mobular*	99	*Mobula japanica* **(*M*. *mobular*)**	100	Spinetail Devil Ray/Manta	FISH1/COI (FB)	MH194482	NO
SF62	2017.02.01	Smooth-hound/Tollo	Fresh filet	Market	Chiclayo (LA)	*Myliobatis chilensis*	99	*Myliobatis chilensis*	99.83	Chilean Eagle Ray/Raya Águila	FISH1/COI (FB)	MH194483	**YES**
SF63	2017.02.01	Smooth-hound/Tollo	Dried	Market	Chiclayo (LA)	*Prionace glauca*	100	*Prionace glauca*	100	Blue Shark/Tiburón Azul	Folm/COI (FB)	MH194484	**YES**
SF64	2017.02.01	Ray/Raya	Dried	Market	Chiclayo (LA)	*Myliobatis chilensis*	99	*Myliobatis chilensis*	99.85	Chilean EagleRay/Raya Águila	Folm/COI (FB)	MH194485	NO
SF65	2017.02.01	Marlin/Merlín	Fresh, whole body	Fish landing site	Cancas (TU)	*Tetrapturus audax* *Tetrapturus audax*	99 99	*Kajikia audax* NO MATCH	100 -	Striped Marlin/Merlín Rayado	FISH1/COI (FB) CR-AG/CR (FB)	MH194486 MK070523	NO
SF66	2017.02.02	Hammerhead Shark/Tollo Martillo	Fresh, whole body	Fish landing site	Cancas (TU)	*Sphyrna zygaena*	100	*Sphyrna zygaena*	100	Smooth Hammerhead/Tiburón Martillo	Folm/COI (FB)	MH194487	NO
SF67	2017.02.09	Electric Ray/Raya Eléctrica	Fresh, whole body	Fish landing site	Cancas (TU)	*Narcine entemedor*	99	*Narcine entemedor*	100	Giant Electric Ray/Raya Eléctrica	Folm/COI (FB)	MH194488	NO
SF68	2017.04.24	Tuna/Atún	Canned, in vegetable oil	Grocery store	Trujillo (LL)	*Thunnus *atlanticus*, *T*. *orientalis*, *T*. *albacares*	98	NO MATCH	-	Tuna/Atún	SH-A/COI (MB)	MH194489	NO
SF69	2017.04.30	Peruvian Sardine/Sardina Peruana, Anchoveta ***egraulis ringens	Canned, in vegetable oil	Supermarket chain	Trujillo (LL)	*Engraulis ringens* *Engraulis ringens*	100 100	*Engraulis ringens* *Engraulis ringens*	100 100	Anchovy/Anchoveta	SH-E/COI (MB) SH-A/COI (MB)	MH194490 MK070518	NO
SF70	2017.04.30	Peruvian Sardine/Sardina Peruana, Anchoveta ***egraulis ringens	Canned, in vegetable oil	Supermarket chain	Trujillo (LL)	*Engraulis ringens* *Engraulis ringens*	100 100	*Engraulis ringens* *Engraulis ringens*	100 100	Anchovy/Anchoveta	SH-E/COI (MB) SH-A/COI (MB)	MH194491 MK070519	NO
SF71	2017.05.20	Peruvian Rock Seabass/Cabrilla	Fish fritters	Restaurant	Chimbote (AN)	*Prionotus stephanophrys*	99	*Prionotus stephanophrys*	99.69	Lumpfish Searobin/Falso Volador, Cabrilla Voladora	FISH1/COI (FB)	MH194492	**YES**
SF72	2017.06.08	Octopus/Pulpo	Grilled	Restaurant	Miraflores (LI)	*Octopus mimus*, *O*. *hubbsorum*	100	*Octopus mimus*	100	Gould Octopus/Pulpo Común	Folm/COI (FB)	MH194493	NO
SF73	2017.06.09	Pacific Menhaden/Machete	Fresh, whole body	Market	Chorrillos (LI)	*Ethmidium maculatum*	99	*Ethmidium maculatum*	100	Pacific Menhaden/Machete	Folm/COI (FB)	MH194494	NO
SF74	2017.06.09	Pacific Menhaden/Machete	Fresh, whole body	Market	Chorrillos (LI)	*Ethmidium maculatum*	99	*Ethmidium maculatum*	99.13	Pacific Menhaden/Machete	Folm/COI (FB)	MH194495	NO
SF75	2017.06.09	Smooth-hound/Tollo de Leche	Refrigerated filet	Supermarket chain	La Molina (LI)	*Mustelus lunulatus*	99	*Mustelus lunulatus*	100	Sicklefin Smooth-hound/Tollo	FISH1/COI (FB)	MH194496	NO
SF76	2017.06.09	Smooth-hound/Tollo de Leche	Refrigerated filet	Supermarket chain	La Molina (LI)	*Mustelus *henlei*, *M*. *californicus* *Mustelus henlei*	98 98	*Mustelus *henlei*, *M*. intermedius *Mustelus *canis*, *M*. *henlei*	98.4 98.2	Brown Smooth-hound/ Tollo	SH-A/COI (MB) SH-E/COI (MB)	MH194497 MK070520	NO
SF77	2017.06.10	Herring/Arenque *Clupea harengus*	Canned. With asparagus sauce. **MSC** certified Imported from Germany	Supermarket chain	La Molina (LI)	*Clupea harengus*	100	NO MATCH	-	Atlantic Herring/Arenque	MiFish/12S (MB)	MH194498	NO
SF78	2017.10.14	Peruvian Rock Seabass/Cabrilla	Fish fritters	Restaurant	Sechura (PI)	*Paralabrax humeralis*	99	*Paralabrax humeralis*	100	Peruvian Rock Seabass/Cabrilla	Folm/COI (FB)	MH194499	NO
SF79	2017.10.14	Peruvian Grunt/Chita	Steamed, whole body	Restaurant	Sechura (PI)	*Anisotremus scapularis*	100	*Anisotremus scapularis*	100	Peruvian Grunt/Chita	FISH1/COI (FB)	MH194500	NO
SF80	2017.10.14	Squid/Calamar	Steamed, rings	Restaurant	Sechura (PI)	*Doryteuthis gahi*	99	*Doryteuthis gahi* **(*Doryteuthis (Amerigo) gahi*)**	99.54	Patagonian Squid/Calamar Común	Folm/COI (FB)	MH194501	NO
SF81	2017.11.09	Octopus/Pulpo	Boiled, in olive sauce	Restaurant	Huarmey (AN)	*Octopus mimus*, *O*. *hubbsorum*	99	*Octopus mimus*	100	Gould Octopus/Pulpo Común	Folm/COI (FB)	MH194502	NO
SF82	2017.11.15	Shrimp/Langostino	Instant noodle soup. Imported from USA	Supermarket chain	Trujillo (LL)	*Metapenaeus dobsoni*	99	*Metapenaeus dobsoni*	99.35	Kadal Shrimp/Langostino	Folm/COI (FB)	MH194503	NO
SF83	2017.12.01	Hammerhead Shark/Tollo Martillo	Fresh, whole body	Fish landing site	Cancas (TU)	*Sphyrna zygaena*	99	*Sphyrna zygaena*	100	Smooth Hammerhead/Tiburón Martillo	FISH1/COI (FB)	MH194504	NO
SF84	2017.12.17	Moonfish/Pez Luna	Fresh filet	Multimarket	Callao (LI)	*Lampris guttatus*	100	*Lampris guttatus*	100	Opah, Moonfish/Pez Luna	COI-3/COI (FB)	MH194505	NO
SF85	2017.12.17	Smooth-hound/Tollo de Leche	Fresh filet	Multimarket	Callao (LI)	*Mustelus henlei*, *M*. *canis*	98	**Mustelus *henlei*	98.15	Brown Smooth-hound/Tollo	COI-3/COI (FB)	MH194506	NO
SF86	2017.12.17	Shortfin Mako/Tollo Diamante	Fresh filet	Multimarket	Callao (LI)	*Isurus oxyrinchus*	100	*Isurus oxyrinchus*	100	Shortfin Mako/Tiburón Diamante	COI-3/COI (FB)	MH194507	NO
SF87	2017.12.17	Basa *Pangasius hypophthalmus*	Filet (vacuum packed). Imported from Vietnam	Supermarket chain	Callao (LI)	*Pangasianodon hypophthalmus*	100	*Pangasianodon hypophthalmus*	100	Striped Pangasius, Swai/Basa	COI-3/COI (FB)	MH194508	NO
SF88	2017.12.17	Sand-Perch/Camotillo, Carajito	Fresh filet	Multimarket	Callao (LI)	*Oreochromis niloticus*, *O*. *aureus*	99	*Oreochromis *niloticus*, *O*. *aureus*	100	Tilapia/Tilapia	COI-3/COI (FB)	MH194509	**YES**
SF89	2017.12.17	Tilapia/Tilapia	Filet (vacuum packed)	Multimarket	Callao (LI)	*Oreochromis mossambicus*, *O*. *niloticus*	100	*Oreochromis *mossambicus*, *O*. *niloticus*	100	Tilapia/Tilapia	COI-3/COI (FB)	MH194510	NO
SF90	2017.12.17	Dolphinfish/Perico	Fish roe	Multimarket	Callao (LI)	*Coryphaena hippurus*	99	*Coryphaena hippurus*	99.85	Dolphinfish/Perico, Dorado	COI-3/COI (FB)	MH194511	NO
SF91	2017.12.17	Dolphinfish/Perico *Coryphaena hippurus*	Fish burger	Supermarket chain	Callao (LI)	*Coryphaena hippurus*	99	*Coryphaena hippurus*	100	Dolphinfish/Perico, Dorado	COI-3/COI (FB)	MH194512	NO
SF92	2017.12.17	Peruvian Sardine and Giant Squid/Sardina Peruana y Calamar Gigante *Engraulis ringens* and *Dosidicus gigas*	Burger	Multimarket	Callao (LI)	*Engraulis ringens*	100	*Engraulis ringens*	100	Anchovy/Anchoveta	COI-3/COI (FB)	MH194513	NO
SF93	2017.12.17	Atlantic Sardine/Sardinas Pilchards del Atlántico Norte	Canned. In spicy sauce. Imported from Chile. Made in Morocco	Supermarket chain	Callao (LI)	*Sardina pilchardus*	100	*Sardina pilchardus*	100	European Pilchard/Sardina Europea	SH-A/COI (MB)	MH194514	NO
SF94	2017.12.17	Bonito/Bonito	Fried filet	Restaurant	Callao (LI)	*Sarda chiliensis*	100	*Sarda chiliensis*	100	Pacific Bonito/Bonito	COI-3/COI (FB)	MH194515	NO
SF95	2017.12.17	Salmon/Salmón	Filet (vacuum packed)	Multimarket	Callao (LI)	*Salmo salar*	100	*Salmo salar*	100	Atlantic Salmon/Salmón	FISH1/COI (FB)	MH194516	NO
SF96	2018.01.08	Atlantic Sardine/Sardinas del Atlántico *Sardina pilchardus*	Canned. With lemon and olive oil. Imported from Spain	Supermarket chain. Delivery system	Callao (LI)	*Sardina pilchardus* *Sardina pilchardus* *Sardina pilchardus*	99 99 100	*Sardina pilchardus* *Sardina pilchardus* NO MATCH	99.56 99.22 -	European Pilchard/Sardina Europea	SH-E/COI (MB) SH-A/COI (MB) MiFish/12S (MB)	MH194517 MK070521 MK070522	NO
SF97	2018.01.12	Peruvian Silverside/Pejerrey	Fried filet, sandwich	Restaurant	Callao (LI)	*Odontesthes regia*	99	*Odontesthes regia*	99.84	Peruvian Silverside/Pejerrey	COI-3/COI (FB)	MH194518	NO
SF98	2018.01.17	Blenny/ Trambollo	Whole body, in parihuela sauce	Restaurant	Callao (LI)	*Labrisomus philippii*	100	*Labrisomus philippii*	100	Chalapo Clinid/Trambollo	FISH1/COI (FB)	MH194519	NO
SF99	2018.01.17	Sailfish/Pez Vela	Filet with Menier sauce	Restaurant	Callao (LI)	*Coryphaena hippurus*	99	*Coryphaena hippurus*	100	Dolphinfish/Perico, Dorado	FISH1/COI (FB)	MH194520	**YES**
SF100	2018.01.17	Sailfish/Pez Vela	Deep fried filet	Restaurant	Callao (LI)	*Coryphaena hippurus*	99	*Coryphaena hippurus*	100	Dolphinfish/Perico, Dorado	FISH1/COI (FB)	MH194521	**YES**
SF101	2018.01.17	Peruvian Grunt/Chita	Fried, whole body, with creamy spicy seafood sauce	Restaurant	Chimbote (AN)	*Anisotremus scapularis*	100	*Anisotremus scapularis*	100	Peruvian Grunt/Chita	FISH1/COI (FB)	MH194522	NO
SF102	2018.01.17	Chiton/Barquillo	Marinated “cebiche”. Sliced	Restaurant	Chimbote (AN)	*Tagelus dombeii*	94	*Tagelus dombeii*	99.83	Jackknife Clam/Navajuela	Folm/COI (FB)	MH194523	**YES**
SF103	2018.01.17	Peruvian Rock Seabass/Cabrilla	Marinated “cebiche”	Restaurant	Chimbote (AN)	*Paralabrax humeralis*	99	*Paralabrax humeralis*	99.69	Peruvian Rock Seabass/Cabrilla	FISH1/COI (FB)	MH194524	NO
SF104	2018.01.17	Sea Snail/Caracol	Marinated “cebiche”	Restaurant	Chimbote (AN)	*Thais chocolata*	98	*Thaisella chocolata*	99.25	Sea Snail/Caracol	Folm/COI (FB)	MH194525	NO
SF105	2018.01.17	Peruvian Grunt/Chita	Fresh, whole body	Market	Chimbote (AN)	*Anisotremus scapularis*	99	*Anisotremus scapularis*	100	Peruvian Grunt/Chita	FISH1/COI (FB)	MH194526	NO
SF106	2018.01.17	Peruvian Grunt/Chita	Fresh, whole body	Market	Chimbote (AN)	*Cheilotrema saturnum*	93	*Cheilotrema fasciatum*	99.85	Arnillo Drum/Burro	FISH1/COI (FB)	MH194527	**YES**
SF107	2018.01.17	Shrimp/Langostino	Fresh, headless	Supermarket chain	Chimbote (AN)	*Litopenaeus vannamei*	100	*Litopenaeus vannamei* **(*Penaeus vannamei)***	100	Whiteleg Shrimp/Langostino	Folm/COI (FB)	MH194528	NO
SF108	2018.01.17	Trout/Trucha	Fresh, whole body	Supermarket chain	Chimbote (AN)	*Oncorhynchus mykiss*	100	*Oncorhynchus mykiss*	100	Rainbow Trout/Trucha Arco Iris	FISH1/COI (FB)	MH194529	NO
SF109	2018.01.17	Seafood Mix	Fresh, cut in small pieces	Market	Chimbote (AN)	*Thais chocolata*	98	*Thaisella chocolata*	99.32	Sea Snail/Caracol	Folm/COI (FB)	MH194530	NO
SF110	2018.01.17	Lumpfish Searobin/Cabrilla Voladora	Fresh, cut in small pieces	Market	Chimbote (AN)	*Prionotus stephanophrys*	100	*Prionotus stephanophrys*	100	Lumpfish Searobin/Cabrilla Voladora	Folm/COI (FB)	MH194531	NO
SF111	2018.01.17	Basa/Basa	Frozen filet	Supermarket chain	Chimbote (AN)	*Pangasianodon hypophthalmus*	99	*Pangasianodon hypophthalmus*	100	Striped Pangasius, Swai/Basa	Folm/COI (FB)	MH194532	NO
SF112	2018.01.17	Seafood Mix	Frozen, cut in small pieces	Supermarket chain	Chimbote (AN)	*Dosidicus gigas*	99	*Dosidicus gigas*	100	Humboldt Squid/Calamar Gigante, Pota	Folm/COI (FB)	MH194533	NO
SF113	2018.01.17	Basa *Pangasius hypophthalmus*	Filet (vacuum packed). Imported from Vietnam	Supermarket chain	Chimbote (AN)	*Pangasianodon hypophthalmus*	99	*Pangasianodon hypophthalmus*	100	Striped Pangasius, Swai/Basa	Folm/COI (FB)	MH194534	NO
SF114	2018.01.17	Dolphinfish/Perico *Coryphaena hippurus*	Fish burger	Supermarket chain	Chimbote (AN)	*Coryphaena hippurus*	99	*Coryphaena hippurus*	100	Dolphinfish/Perico, Dorado	Folm/COI (FB)	MH194535	NO
SF115	2018.01.17	Crab Meat/Pulpa de Cangrejo *Cancer setosus*	Frozen meat	Supermarket chain	Chimbote (AN)	*Romaleon polyodon*	100	*Romaleon polyodon* **(*R*. *setosum*)**	100	Hairy Crab/Cangrejo Peludo	Folm/COI (FB)	MH194536	NO
SF116	2018.01.17	Peruvian Sardine and Giant Squid/Sardina Peruana y Calamar Gigante *Engraulis ringens* and *Dosidicus gigas*	Burger	Supermarket chain	Chimbote (AN)	*Engraulis ringens*	100	*Engraulis ringens*	100	Anchovy/Anchoveta	Folm/COI (FB)	MH194537	NO
SF117	2018.01.23	Clam/Almeja	Fresh, whole body	Market	Chimbote (AN)	*Semele *solida*	92	NO MATCH	-	Clam/Almeja	Folm/COI (FB)	MH194538	NO
SF118	2018.01.23	Surf Clam/Macha	Fresh, cut in small pieces	Market	Chimbote (AN)	*Tagelus dombeii*	94	*Tagelus dombeii*	99.47	Jackknife Clam/Navajuela	Folm/COI (FB)	MH194539	**YES**
SF119	2018.01.23	Surf Clam/Maruchita	Fresh, whole body	Market	Chimbote (AN)	*Donax obesulus*	99	NO MATCH	-	Surf Clam/Maruchita, Palabrita	Folm/COI (FB)	MH194540	NO
SF120	2018.02.06	Sand-Perch/Camotillo	Marinated “cebiche”	Restaurant	Chimbote (AN)	*Paralabrax humeralis*	100	*Paralabrax humeralis*	100	Peruvian Rock Seabass/Cabrilla	FISH1/COI (FB)	MH194541	**YES**
SF121	2018.02.10	Sailfish/Pez Vela	Fried filet, with creamy spicy seafood sauce	Restaurant	Callao (LI)	*Coryphaena hippurus*	99	*Coryphaena hippurus*	100	Dolphinfish/Perico, Dorado	FISH1/COI (FB)	MH194542	**YES**
SF122	2018.02.17	Grouper/Mero	Fish meat in Parihuela sauce	Restaurant	San Isidro (LI)	*Mycteroperca microlepis*	94	*Mycteroperca xenarcha*	100	Broomtail Grouper/Mero Negro	FISH1/COI (FB)	MH194543	NO
SF123	2018.02.17	Peruvian Scallop/Concha de Abanico	Seafood rice	Restaurant	San Isidro (LI)	*Argopecten purpuratus*	99	NO MATCH	-	Peruvian Scallop/Concha de Abanico	Pect16/16S (FB)	MH194544	NO
SF124	2018.02.17	Octopus/Pulpo	Seafood rice	Restaurant	San Isidro (LI)	*Octopus hubbsorum*, *O*. *mimus*	99	*Octopus mimus*	100	Gould Octopus/Pulpo Común	Folm/COI (FB)	MH194545	NO
SF125	2018.02.19	Almaco Jack/Fortuno	Marinated “cebiche”	Restaurant	Miraflores (LI)	*Seriola rivoliana*	99	*Seriola rivoliana*	99.82	Almaco Jack/Fortuno	FISH1/COI (FB)	MH194546	NO
SF126	2018.02.20	Barracuda/Picuda Norteña	Fresh, whole body	Multimarket	Callao (LI)	*Sphyraena ensis*	100	*Sphyraena ensis*	100	Barracuda/Picuda	FISH1/COI (FB)	MH194547	NO
SF127	2018.02.26	Surf Clam/Macha	Boiled tongues	Multimarket	Callao (LI)	*Tagelus dombeii*	94	*Tagelus dombeii*	100	Jackknife Clam/Navajuela	COI-ALT/COI (FB)	MH194548	**YES**
SF128	2018.02.26	Surf Clam/Palabrita	Whole body. Salted	Multimarket	Callao (LI)	*Donax obesulus*	100	NO MATCH	-	Surf Clam/Maruchita, Palabrita	Folm/COI (FB)	MH194549	NO
SF129	2018.03.03	Peruvian Silverside/Pejerrey	Fresh filet with chili	Restaurant	Miraflores (LI)	*Odontesthes regia*	99	*Odontesthes regia*	100	Peruvian Silverside/Pejerrey	FISH1/COI (FB)	MH194550	NO
SF130	2018.03.03	Cachema Weakfish/Charela	Stir-fried	Restaurant	Miraflores (LI)	*Coryphaena hippurus*	100	*Coryphaena hippurus*	100	Dolphinfish/Perico, Dorado	FISH1/COI (FB)	MK070511	**YES**
SF131	2018.03.18	Sailfish/Pez Vela	Frozen filet	Multimarket	Callao (LI)	*Kajikia albida* *Kajikia albida*	99 99	*Kajikia albida* NO MATCH	100 -	Atlantic White Marlin/Merlín Blanco	FISH1/COI (FB) CR-AG/CR (FB)	MH194551 MK070524	**YES**

* Identification to the genus level (ID% or Similarity% <98)

** Identification to the genus level after phylogenetic analysis

*** A misspelled scientific name was written on label

Locations: TU Tumbes, PI Piura, LA Lambayeque, LL La Libertad, AN Ancash, and LI Lima

Primer sets: FISH1: FishF1 and FishR1 [37], Folm: LCO1490 and HCO2198 [17], SH-A: Fish_miniA_F_t and Fish_miniA_R_t [23], SH-D: Fish_miniD_F_t and Fish_miniD_R_t [23], SH-E: Fish_miniE_F_t and Fish_miniE_R_t [23], Pect16: Pect16BCF and Pect16BCR [18], COI-3: “cocktail” primer set VF2_t1, FishF2_t1, FishR2_t1, and Fr1d_t1 [38], MiFish: MiFish-U-F and MiFish-U-R [21], COI-ALT: COIF-ALT and COIR-ALT [39], CR-AG: A and G [40]

Marker: COI cytochrome c oxidase subunit I, 16S ribosomal RNA, 12S ribosomal RNA, CR control region

FB: full barcode, MB: mini-barcode

#### Fish landing sites, wholesale fish markets, and markets

A total of 51 samples were identified from FLSs (n = 21), WFM (n = 7) and MKs (n = 23), represented mainly by batoids (72%), sharks (100%), and bony fishes (35%), respectively (see S1 Fig). Among batoid specimens (rays and their relatives) collected from FLSs (n = 15) and MKs (n = 7), including one Chilean eagle ray specimen labeled as smooth-hound, we identified nine species belonging to seven families: Aetobatidae, Arhynchobatidae, Dasyatidae, Mobulidae, Myliobatidae, Narcinidae, and Rhinobatidae (Table 2); and one genus (*Gymnura* sp.) from Gymnuridae (see S3 Appendix). Three (*Hypanus dipterurus*, *Myliobatis longirostris*, and *Sympterygia brevicaudata*) of the nine identified species collected from different FLSs in TU (collection date January 2017) were not found in recent landing reports (from January 2014 to July 2018) from Tumbes (C. Luque, personal communication). Besides, the sample identified as shorttail fanskate *S*. *brevicaudata* (SF47, BOLD similarity 100%) was labeled as witch skate *Rostroraja velezi*; the latter species was included in landing records from Tumbes (C. Luque, personal communication). These findings highlight the necessity for implementation of periodic genetic monitoring programs across landing sites to support fisheries management and conservation efforts of batoid species.

In 2016, total “ray” landings from Peru reached 2440 metric tonnes (MT), with 72.34% of this production (1765 MT) destined to the domestic fresh fish market, while the remaining was used for cured fish production [8]. The giant devil ray *M*. *mobular* (Myliobatiformes: Mobulidae), which is the most landed mobulid species in northern Peru [49], was also the most abundant batoid and only mobulid species detected in this study, being found in three FLSs (n = 5), three MKs (n = 4), and one RT (n = 1). In Peru, the genus *Mobula* comprises five species including *M*. *birostris* (formerly *Manta birostris*), *M*. *tarapacana*, *M*. *mobular* (formerly *M*. *japanica* [47]), *M*. *thurstoni*, and *M*. *munkiana* [50]. Predominantly gillnets are used by small-scale and industrial fisheries to target mobulids, but they are also caught as bycatch in the tuna purse seine fishery [49]. In spite of their commercial value, conservation concerns, and promising management and conservation efforts targeting chondrichthyan species (i.e., PAN Tiburón-Perú and a law prohibiting *M*. *birostris* fishery, see S4 Appendix and S5 Appendix), there have not been any molecular studies on Peruvian marine rays. Our results could be used to better understand the diversity of commercially important rays, providing baseline data for further genetic studies necessary to design and implement conservation actions.

The smooth hammerhead *Sphyrna zygaena* (Carcharhiniformes: Sphyrnidae), which is the third most commonly landed shark species in Peru [51], was found at the three retailer categories described in this section (FLS, WFM, and MK), where it was usually labeled as thresher shark. S4 Table shows all identified samples grouped by retailer category and seafood type. Nine samples were identified as *S*. *zygaena* with MB and FB (BOLD similarity 100%). Of particular concern was the detection of six specimens collected during closed season (January 1 to March 10, RM N° 008-2016-PRODUCE), including five headless samples (SF50 to SF54 from WFM-LL) labeled as thresher sharks (*Alopias* sp.) and one sample (SF66 from FLS-TU) landed as whole body. Illegal, unreported and unregulated (IUU) fishing can be profitable due to the high demand for overexploited and protected species, and low risk of getting caught or being punished, especially when it takes place in countries where enforcement is weak [52]. *Sphyrna zygaena* is categorized as “Vulnerable” by the IUCN Red List of Threatened Species. Regrettably, the shark fishery in Peru is poorly regulated and monitored, mainly because fisheries managers put more effort in controlling small pelagic resources which dominate the fishing industry [34].

Other interesting findings at FLSs were the landings of one whale shark specimen (SF46) *Rhincodon typus* (Orectolobiformes: Rhincodontidae) and one sample (SF65) identified as striped marlin *Kajikia audax* (Perciformes: Istiophoridae), both as whole bodies. The commercial fishing of both species has been banned by the Peruvian government (see text in S4 Appendix and S6 Appendix). The whale shark DNA sequence obtained herein represents the first publically available nucleotide sequence of *R*. *typus* (GenBank accession number MH194467) caught in Peruvian waters, which could be useful for further comparative studies, therefore the specimen tissue and DNA from sample SF46 are available upon request.

Among bony fishes (class Actinopterygii) collected from MKs, the economically valuable genus *Anisotremus* (Perciformes: Haemulidae) was represented by two species: the Peruvian grunt *A*. *scapularis* and the burrito grunt *A*. *interruptus*. In one MK from Ancash, we bought a bag containing 10 fish specimens labeled as “Peruvian grunt”, however one specimen was larger and darker than the others. Molecular analysis showed that the “dark grunt” (SF106) was actually arnillo drum *Cheilotrema fasciatum* (family Sciaenidae), which strongly resemble grunts in appearance but is of lower economic value. Another sample (SF105) from the same bag was identified as the Peruvian grunt *A*. *scapularis*. Apparently, the arnillo drum, larger in size, was put there to increase the total product weight.

#### Grocery store, supermarket chains, and multimarket

A total of 35 samples were identified from SMCs (n = 22), MM (n = 12), and a GS (n = 1), (S4 Table). The only sample collected from GS was a canned tuna (SF68) identified as *Thunnus* sp. The most abundant group detected in both SMCs and MM was Actinopterygii with 55% and 66%, respectively (S1 Fig). Among the samples collected from SMCs and MM, the Peruvian anchovy *E*. *ringens* (canned and fish burger presentations), dolphinfish *C*. *hippurus* (fish burger and fish roe presentations), and Humboldt squid *D*. *gigas* (seafood mix presentation) represented some of the most important species from landings for direct human consumption during the year 2016 [8]. Two canned anchovy products (SF69 and SF70, SMC at LL) labeled as “Peruvian sardine” were identified as *E*. *ringens*. In 2009, the Ministry of Production of Peru, aiming to promote internal consumption of anchovy as well as to conquer new international markets, adopted the name “Peruvian sardines” for processed (i.e., canned) Peruvian anchovies [53]. This marketing strategy is due to the fact that in the international market, sardine is usually in higher demand than anchovy [54]. However, the presence of the local sardine species *Sardinops sagax* also known as “Peruvian sardine” [55] may cause confusion among local consumers.

Imported items, representing both farmed and wild species, were found only in SMC and MM retailers. During 2017, Peru imported 145344 MT of seafood products valued at US$306 million; frozen and canned products accounted for 70% of the total [56]. We authenticated nine imported seafood products belonging to six species from five orders including Perciformes (*Kajikia albida*, frozen filet n = 1), Clupeiformes (*Sardina pilchardus* and *Clupea harengus*, canned n = 3), Salmoniformes (*Salmo salar*, vacuum packed filet n = 1), Siluriformes (*Pangasianodon hypophthalmus*, frozen and vacuum packed filets n = 3), and Decapoda (*Metapenaeus dobsoni*, instant noodle soup n = 1). In 2015, Chilean port authorities detected a shipment (valued at US$19 million) from Callao (Peru) of 37200 cans of Pacific menhaden (*Ethmidium maculatum*) labeled as horse mackerel (*Trachurus murphyi*) [57]. In this regard, the use of DNA-based technologies for seafood authentication is imperative to ensure proper label information, not only for domestic and imported products but also for Peruvian exports.

Interestingly, in one supermarket from Lima, we found different imported canned products carrying the Marine Stewardship Council (MSC) blue ecolabel. The MSC is an international non-profit organization that sets a standard (MSC Fishery Standard) used to assess sustainable fisheries all over the world. Currently, there are no Peruvian fisheries holding MSC certification or undergoing full assessment. In 2015, a molecular barcoding test of a total of 256 MSC labeled products (from 16 countries, covering 13 fish species) performed by an independent laboratory revealed that 99.6% were correctly labeled [58]. Herein, we were able to verify the correct species information from one MSC certified canned herring sample imported from Germany and labeled as *Clupea harengus* (SF77, bought in SMC-LI) using the 12S rRNA gene eDNA metabarcoding primer set MiFish-U designed by Miya et al. [21].

We detected only one case of mislabeling in an SMC (from LL) in which a filet sample labeled as guitarfish (SF58) was determined to actually be Chilean eagle ray *M*. *chilensis* (BOLD similarity 100%). However, it is difficult to determine whether it was an intentional case of mislabeling due to the fact that SMCs usually rely on wholesale seafood distributors. It is important to mention that SMCs employ trained personnel and safety protocols including the application of cold chain management systems, thus preserving food quality and ensuring food safety. However, the aforementioned practices make SMC seafood products more expensive than those of popular MKs; sometimes the price difference is as high as 300% [59].

Three smooth-hound filet samples (SF56, SF57, and SF85) were first identified as *Mustelus henlei* (BOLD similarity 98.15–98.55%). However, NJ and BI phylogenetic analyses (Fig A in S2 Appendix) clustered those samples in a unique clade with high nodal support. Therefore, samples SF56, SF57, and SF85 were assigned to *Mustelus* sp. Another smooth-hound filet sample (SF75, SMC-LI) was identified as *Mustelus lunulatus* (BOLD similarity 100%). In Peru, smooth-hounds (*Mustelus* spp.), houndsharks (*Triakis* spp.), and catsharks (*Schroederichthys* spp.) are usually reported under the same common name “*tollo*” ([34], FISHLANDING-2017 list). Peruvian shark landing statistics at species level include only three *Mustelus* species: *M*. *whitneyi*, *M*. *mento*, and *M*. *dorsalis* [51]. A reduced frequency in landing occurrences, combined with low taxonomic resolution at landing sites and a poorly regulated and monitored fishery [34], may have been masking or “diluting” the presence of *M*. *lunulatus* from landing reports. However, we cannot rule out the possibility that sample SF75 was imported from Ecuador (where *M*. *lunulatus* also occurs), which is an important source of shark imports to Peru [51].

Inaccurate identification of morphologically similar species in combination with poor taxonomic resolution of fisheries landing reports and the application of inaccurate names will not only cause considerable economic impacts but also lead to undesired consequences for fishery management [60] including local population depletion. *M*. *lunulatus* is not included in the “Identification guide to commercially important sharks from Peru” [61], which is a field guide for identifying most frequent shark species in landings of artisanal fisheries of Peru. In this regard, government fishery officers must undergo training in detecting not only main commercial species but also the ones that are infrequently landed. Field identification guides should consider including the “less commercial” species.

One surprising result was the mislabeling of tilapia *Oreochromis* sp. as “sand-perch” (SF88, collected from MM, see Fig 1D). Passing off cheap farmed tilapia as more expensive wild fish has been reported in previous studies [62, 63]. During 2013, frozen imports of tilapia from China accounted for more than 50% of total domestic market sales [64]. China is Peru’s biggest trade partner, with investments exceeding US$14.00 billion [65]. A Free Trade Agreement (FTA) between Peru and China was signed on April 28, 2009, and entered into force on March 1, 2010 [66] bringing valuable opportunities for Peruvian entrepreneurs to go through Chinese markets duty-free. Unfortunately, imported Chinese tilapia enters the Peruvian market at significantly lower prices than locally produced ones [64].

Shellfish accounted for 18% of SMC samples, comprising three crustacean (14%) (*M*. *dobsoni*, *P*. *vannamei*, and *Romaleon setosum*), and one mollusk (4%) species (Humboldt squid *D*. *gigas*). Only mollusks (17%) were collected from MM, represented by two bivalve species (*Tagelus dombeii* and *Donax obesulus*). Two shucked shellfish samples (SF118 from MK-AN and SF127 from MM-LI) labeled as surf clam “*macha*” (*Mesodesma donacium*) were identified as jackknife clam *T*. *dombeii* (BOLD similarity 99.47–100%). Peruvian populations of the surf clam *M*. *donacium* have been depleted and its fishery abruptly collapsed, mainly due to the combined effects of unregulated overexploitation and adverse climatic events (El Niño/Southern Oscillation-ENSO) [67]. As evidenced by our results, the great demand that still exists for this bivalve species makes this resource vulnerable to substitution by other species in different Peruvian cities.

#### Restaurants

The results of the present study provide a snapshot of species availability in some seafood restaurants. Forty-five samples were identified covering 15 bony fishes, nine shellfish, three sharks, and one batoid species (S4 Table). Within the bony fishes, which represented 60% of restaurant samples (S1 Fig), we identified high market-value species such as flounder (Paralichthyidae), grunts (Haemulidae), and grouper and seabass (Serranidae). In Peru, groupers (*Epinephelus* spp. and *Mycteroperca* spp.) and grape-eye seabass (*Hemilutjanus macrophthalmos*) are considered “luxury” seafood species and in high demand, which makes them more prone not only to overexploitation but also to substitution by cheaper ones. Two restaurant samples (SF7 and SF17) labeled as grouper were identified as South Pacific hake *M*. *gayi* and dolphinfish *C*. *hippurus*, respectively. Similarly, two samples (SF8 and SF9) labeled as grape-eye seabass were found to be swordfish *Xiphias gladius*.

When mislabeling occurs on board or at landing, the error continues along the food chain to the consumer [68]. Consequently, final seafood retailers such as restaurants are more vulnerable to receive mislabeled products. Herein, 17 (38%) of the 45 identified samples bought from 21 restaurants across 10 different districts were molecularly identified as different species to those declared by the restaurant staff or menu list (S3 Table). Similar substitution levels (from 26 to 50%) of samples collected in restaurants have been reported in other studies [13, 69, 70]. We want to emphasize, however, that mislabeling should not always be considered as fraud. Instead, it could be the result of species misidentification, due to the confusion generated by the use of different vernacular names in different regions or countries, or when mistakes occur during product information management by mid-chain players. Fortunately, many restaurateurs view seafood sustainability as a requisite for future viability, and some remarkable initiatives have been undertaken by Peruvian chefs engaging directly with artisanal fishers [71, 72].

Our results revealed that in cases of mislabeling, the species most commonly used as a replacement was dolphinfish (*C*. *hippurus*), which was served as grouper (SF17), corvine (SF23), sailfish (SF99, SF100, SF121), and cachema weakfish (SF130) in five different restaurants from La Libertad and Lima. The fact that dolphinfish meat is being used in some restaurants to replace other “white flesh” species including corvine drum was reported in a previous study [73] based on visual inspections (R. Gozzer, personal communication). The dolphinfish’s white flesh makes this species a potential substitute for others high-priced species. Peru is the main producer of dolphinfish with estimated landings accounting for more than 50% of global catches [73]. Peruvian dolphinfish fishery is targeted exclusively by the artisanal fleet, representing one of the nation’s most important artisanal fishery; however it is poorly regulated with high levels of informality along its supply chain [73].

Eight shellfish species including mollusks (octopuses, squids, scallops, mangrove cockle, and sea snail) and crustaceans (crab and shrimp) were identified from restaurant samples (S4 Table). Two species (whiteleg shrimp *P*. *vannamei* and Peruvian scallop *A*. *purpuratus*) account for most of Peruvian mariculture production [8]. Both are well known and widely accepted by consumers, becoming a target product for most seafood restaurants. In Peru, shrimp production has been growing steadily at about 10 percent annually since 2008 [56]. In 2017, shrimp production reached 26768 MT, of which 80% (21400 MT, valued at US$164.1 million) was exported [56]. On the other hand, Peruvian scallop production has decreased significantly from 67694 MT in 2013 to 13137 MT in 2017. An estimated 3300 MT (valued at US$54.3 million) was exported in 2017 [56]. The decline in scallop production was driven mainly by the “coastal El Niño”, which affected up to 98% of production in northern Peru during 2016–17 [74]. This drop in Peruvian scallop production has affected not only the domestic market but also global scallop trade [75].

Another valuable shellfish species is the Gould octopus *Octopus mimus* (Octopoda: Octopodidae), which supports an important artisanal fishery in Peru. Landing estimates were 5405 MT in 2016 [8]. A genetic study has suggested the possible conspecificity between *O*. *mimus* and the Hubb’s octopus *O*. *hubbsorum* [76]. The distribution of *O*. *mimus* is believed to be restricted from northern Peru to Chile, whereas *O*. *hubbsorum* is found from the Gulf of California to Oaxaca in Mexico [76]. However, some molecular studies have reported the presence of *O*. *mimus* in Central America and Ecuador [76, 77] (and references therein).

**Fig 2:**
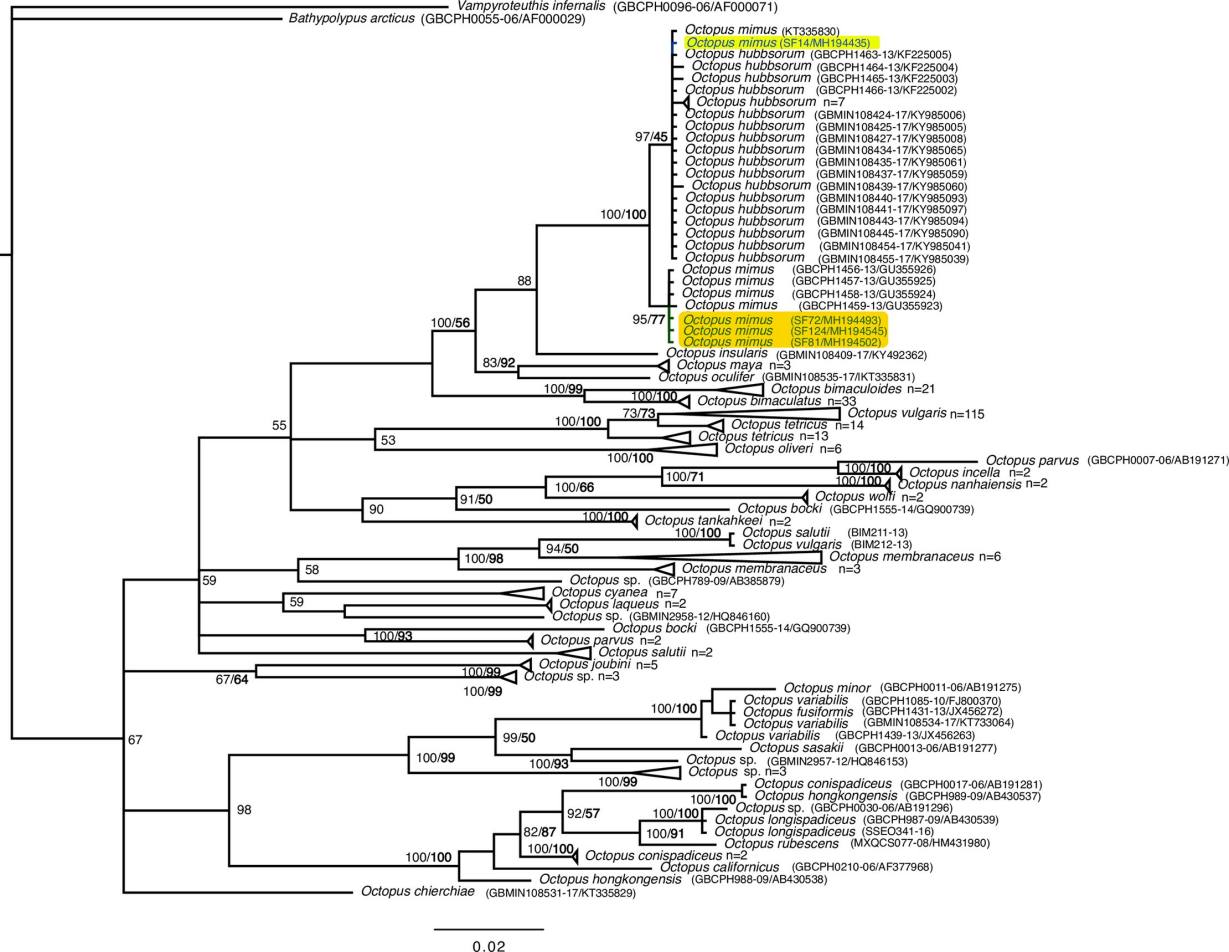
Phylogenetic tree based on Bayesian inference (BI) and Neighbor-Joining (NJ) for the identification of samples SF14, SF72, SF81, and SF124 *Octopus mimus*. Phylogenetic tree based on COI barcode sequences (576 bp) from samples SF14, SF72, SF81, and SF124 (*Octopus mimus*, this study) and other *Octopus* reference sequences available in BOLD and NCBI. Sample SF14 is highlighted in blue and shaded in yellow. Samples SF72, SF81, and SF124 are highlighted in green and shaded in orange. Bayesian consensus tree was inferred with five million generations under the GTR+I+G substitution model. NJ tree was constructed with 1000 bootstrap replicates under the Kimura-2-parameter (K2P) model. Nodal supports for Bayesian inference posterior probabilities and bootstrap values for NJ analysis (highlighted in bold) above 45% are shown. Samples from this study include identification code and GenBank accession numbers. Reference sequence labels include BOLD process ID and GenBank accession numbers. Vampire squid *Vampyroteuthis infernalis* and North Atlantic octopus *Bathypolypus arcticus* were used as outgroup.

We analyzed four octopus samples collected from RTs in LL, AN, and LI. Barcoding results matched samples SF72, SF81, and SF124 to *O*. *mimus* and sample SF14 to *O*. *hubbsorum* (BOLD similarity 100%). Our phylogenetic results (Fig 2) showed congruent topologies between BI and NJ trees, with samples SF72, SF81, and SF124 clustered within the *O*. *mimus* subclade (BA posterior probability 95%, NJ bootstrap support 77%) with a maximum of 0.2% (K2P) within-cluster divergence, while SF14 was within the *O*. *hubbsorum* subclade (BA posterior probability 97%, NJ bootstrap support 45%) showing a maximum within-cluster divergence of 0.3% (K2P). The minimum genetic distance (K2P) between both subclades was 0.7%. Interestingly, sample SF14 shares the same haplotype with the *O*. *mimus* specimen reported in Ecuador (GenBank accession KT335830) [77]. Data mined from customs information imports from the National Customs Superintendency of Peru (SUNAT) (http://www.aduanet.gob.pe/cl-ad-itconsultadwh/ieITS01Alias?accion=consultar&CG_consulta=2), showed that recent octopus imports were represented only by *O*. *mimus* from Chile (from 2014 to 2017) and *O*. *vulgaris* from the Philippines in 2014. Thus, without further evidence of *O*. *hubbsorum* imports or a recent range expansion towards the South Pacific, we assigned sample SF14 to *Octopus mimus*. Further studies must be carried out to solve the taxonomic status of the economically important *O*. *mimus*, which is still under debate.

### Mislabeling

Overall, 35 (26.72%) out of the 131 identified samples were found to be mislabeled, the majority came from markets and restaurants. As expected, most (94.28%) of the misrepresented samples were processed or cooked, where morphological features had been altered or removed. Mislabeled samples included 15 bony fishes (42.86%), 13 sharks (37.14%), four batoids (11.43%), and three mollusk (8.57%) specimens. S3 Table summarizes all mislabeled samples detected in each retailer category. Except for grocery store (GS), where only one sample was collected, we detected at least one mislabeling in each sampling site: FLS (n = 1, 4.76%), WFM (n = 5, 71.42%), MK (n = 8, 34.78%), SMC (n = 1, 4.54%), MM (n = 3, 25%), and RT (n = 17, 37.77%). However, it must be mentioned that sample sizes from WFM (n = 7) and GS (n = 1) were not representative, which prevented us from making any meaningful inferences on mislabeling rates from those retailer categories.

Nevertheless, our results could be used as a starting point to identify the major mislabeled species and the most common substitute species, as well as high priority stages for species substitution control along the supply chain. For example, among chondrichthyans, the most used species was the Chilean eagle ray *M*. *chilensis*, which was incorrectly labeled as manta (SF29-MK), guitarfish (SF58-SMC), and smooth-hound (SF62-MK). A high mislabeling rate (54.17%) was found among all shark samples (n = 24) collected across six different stages of the supply chain, with a total of 13 mislabeling cases involving two “Vulnerable” (*S*. *zygaena* and *A*. *pelagicus*) and one “Near Threatened” (*Prionace glauca*) species. This result could be a consequence of inaccurate species identification practices at early stages of the supply chain coupled with weak enforcement of shark regulations within the seafood sector. In Peru, the shark fishery is poorly monitored, worsened by the superficial taxonomic identification at landing sites across the country [34]. The bony fish most commonly used to replace other species was dolphinfish *C*. *hippurus*, which was served as grouper, corvine, sailfish, and cachema weakfish in six cases involving restaurant samples. Our results indicate that mislabeling is a common issue within the Peruvian seafood sector. Markets and restaurants accounted for the most cases of mislabeling, making those retailer categories potential candidates to be considered as priority control stages. However, further studies covering wider geographical areas with larger sample sizes from each supply chain stage are needed to support our mislabeling results.

The use of the same common name for different species or a single species having different vernacular names even within the same region or country is a common issue in seafood labeling [78]. In Peru, seafood commercial names used for more than one species include “*mero*” (e.g., groupers *Epinephelus* spp., *Mycteroperca* spp., *Alphestes* spp.), “*lenguado*” (e.g., flounders *Paralichthys* spp., *Etropus* spp.), “*tollo*” (e.g., smooth-hounds *Mustelus* spp., *Triakis* spp., catsharks *Schroederichthys* spp.), “*ojo de uva*” (i.e., grape-eye seabass *Hemilutjanus macrophthalmos* and mocosa ruff *Schedophilus haedrichi*), “*almeja*” (e.g., clams *Semele* spp., *Gari* spp.), and “*barquillo*” (e.g., *Chiton* spp., *Acanthopleura* spp.), just to mention a few. Despite this large number, few efforts have been made to regulate the application of standardized Peruvian commercial fish names [79]. To standardize the nomenclature used for seafood products, official guides with acceptable market names were published by the US Food and Drug Administration (FDA) in 1988 [80] and the European Union (EU) in 2001 [81]. To avoid ambiguities with accepted market names within the Peruvian seafood sector, the creation of an official standardized list of commercial fish names is strongly encouraged.

### Conservation status and regulatory framework

A revision of the conservation status using the IUCN Red List of Threatened Species [82] showed that among all identified species, four samples belonged to species classified as “Endangered”: *R*. *typus* (n = 1) and *P*. *hypophthalmus* (n = 3); 13 shark and one marlin samples came from four species classified as “Vulnerable”: *S*. *zygaena* (n = 9), *Isurus oxyrinchus* (n = 1), *A*. *pelagicus* (n = 3), and *K*. *albida* (n = 1); and seven samples came from three species listed as “Near Threatened”: *K*. *audax* (n = 1), *P*. *glauca* (n = 5), and *M*. *longirostris* (n = 1). We should mention that the Endangered *P*. *hypophthalmus* production come from large-scale farms. The remaining identified samples to the species level (n = 86, 65.65%) correspond to species listed as “Least Concern” (n = 44), “Not Evaluated” (n = 23), and “Data Deficient” (n = 19).

As aforementioned, *M*. *japanica* and *M*. *mobular* belong to the same species [47], with nomenclatural priority given to *M*. *mobular* [83]. *Mobula japanica* was considered a wide-ranging circumtropical species assessed as “Near Threatened”, whereas *M*. *mobular* was considered a Mediterranean endemic with a Red List Assessment of “Endangered” [84]. The lumping of both species represents a change in “taxonomic concept” requiring a reassessment for the Red List, which is scheduled as part of the Global Shark Trend’s pelagic species project in 2018 [84].

In Peru, the Ministry of Production (PRODUCE) through its Vice-ministry of Fisheries is the entity responsible for the establishment and application of fisheries management regulations. The legal framework that regulates fishing activities aiming to ensure preservation and the sustainable exploitation of the aquatic resources comprises the General Fisheries Act (DL N° 25977), its Regulations on the General Fisheries Act (DS N° 012-2001-PRODUCE, modified by DS N° 015-2007-PRODUCE), and the Control Regulation and Sanction of the Fishing and Aquaculture Activities (DS N° 017-2017-PRODUCE).

A summary of the most important and recent regulations related to the three threatened species groups detected in this study (i.e., sharks, mobulids, and istiophorids) is presented in S4 Appendix, S5 Appendix, and S6 Appendix. The regulatory framework related to labeling of manufactured products detected herein is described in S7 Appendix.

## Conclusions

This study represents the first attempt to assess the biodiversity present across different stages of the Peruvian supply chain using full and mini DNA barcoding, providing baseline data on the incidence of major mislabeled and the most common substitute species within the Peruvian seafood sector. Our results showed that full and mini-barcoding approaches are reliable and useful tools for species diversity determination, authentication and mislabeling detection of seafood products traded in the Peruvian market, which includes a wide range of taxonomic groups. A current drawback is the lack of barcoding reference sequences of some economically important Peruvian seafood species including smooth-hounds *M*. *dorsalis* and *M*. *whitneyi*, and butterfly ray *Gymnura afuerae*. In this light, the generation of a comprehensive Peruvian seafood barcoding library based on a mass genetic profiling of seafood biodiversity will be helpful to overcome these disadvantages. A major effort on seafood traceability must be undertaken by governmental agencies, fishery policy makers, and scientists to protect treasured marine species such as those on the IUCN Red List (e.g., endangered whale shark and vulnerable hammerhead shark) and to detect illegal fishing during closed seasons. The molecular evidence presented in this study suggests that illegal, unreported and unregulated (IUU) fishing activities are occurring in some areas of the Peruvian seafood sector as well as fraudulent actions within the supply chain. Peruvian artisanal fisheries lack of basic information for their proper management, with no good records on commercial fisheries landings, and no proper monitoring of seafood along the supply chain [73]. Albeit illegal incidental or opportunistic catches of threatened marine species have been already reported by Peruvian governmental inspectors and researchers, that is, however, only the tip of the iceberg, compared with what is really slipping through the net. Action plans for implementing standard and emerging DNA technologies, including rapid molecular detection techniques and environmental DNA (eDNA) to monitor endangered and heavily exploited species must be a priority concern. To strengthen traceability, strict enforcement of fish inspection programs based on DNA barcoding throughout the seafood industry and retailers must be conducted by government agencies. DNA barcoding will help to prevent and combat illegal or “pirate” fishing, especially in a mega-diverse country with high fish consumption such as Peru.

## Supporting information

S1 Table PCR mix composition and amplification conditions for full and mini-barcoding primer sets using two different commercial master mix brands. PCR and sequencing primers are indicated.
(XLSX)

S2 Table PCR and sequencing efficiency of mini-barcoding primers. PCR Polymerase chain reaction, SEQ Sequencing, P Positive result, N Negative result, MB mini-barcoding, FB fullbarcoding.
(XLSX)

S3 Table Summary list of all mislabeled samples. Mislabeled samples are grouped by retailer category and location.
(XLSX)

S4 Table Molecular identification of 131 seafood samples. Samples are grouped by origin (retailer category) seafood type, and retailer location. Samples code highlighted in bold and denoted with an asterisk (*) correspond to mislabeled samples. English/Spanish ("declared as"), identification results from BLAST and BOLD analysis, GenBank accession numbers and nucleotide consensus sequences generated in this study are given for each sample. Genera highlighted in bold correspond to samples identified to the genus level.
(XLSX)

S1 Fig Pie charts depicting percentage of seafood type contributions in each retailer category. Wholesale fish markets (WFM, n = 7, only shark samples) and grocery store (GS, n = 1, tuna sample) categories are not included.
(PDF)

S1 Appendix Phylogenetic identification results of samples SF65 striped marlin *Kajikia audax* and SF131 Atlantic white marlin *K*. *albida*. (PDF)

S2 Appendix Phylogenetic identification results of samples SF56, SF57, and SF85 smooth-hound *Mustelus* sp. (PDF)

S3 Appendix Phylogenetic identification results of samples SF42 and SF44 butterfly ray *Gymnura* sp. (PDF)

S4 Appendix Conservation status and regulatory framework of sharks. (PDF)

S5 Appendix Conservation status and regulatory framework of mobulids. (PDF)

S6 Appendix Conservation status and regulatory framework of istiophorids. (PDF)

S7 Appendix Regulatory framework related to labeling of manufactured products. (PDF)
