# Supplementary material for: A glimpse into the genetic diversity of the Peruvian seafood sector: Unveiling species substitution, mislabeling and trade of threatened species
Source: PLoS One. 2018 Nov 16;13(11):e0206596. doi: 10.1371/journal.pone.0206596 (PMC6239289; doi:10.1371/journal.pone.0206596)
Supplement: S1 Fig — Wholesale fish markets (WFM, n = 7, only shark samples) and grocery store (GS, n = 1, tuna sample) categories are not included. (PDF) [file pone.0206596.s005.pdf]

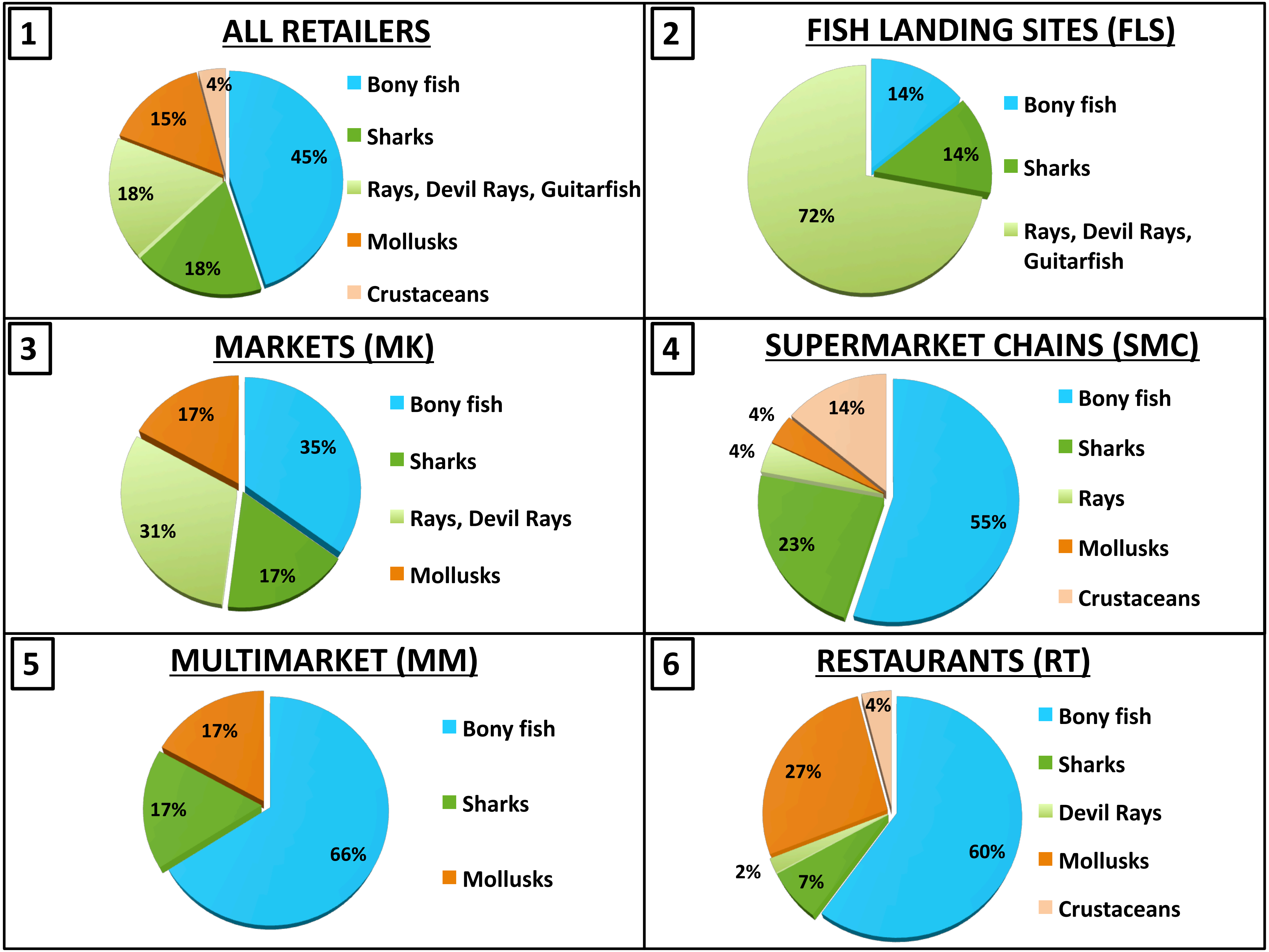
1
ALL RETAILERS
4%
15%
45%
18%
18%
Bony fish
Sharks
Rays, Devil Rays, Guitarfish
Mollusks
Crustaceans
2
FISH LANDING SITES (FLS)
14%
14%
72%
Bony fish
Sharks
Rays, Devil Rays, Guitarfish
3
MARKETS (MK)
17%
35%
31%
17%
Bony fish
Sharks
Rays, Devil Rays
Mollusks
4
SUPERMARKET CHAINS (SMC)
4%
14%
4%
55%
23%
Bony fish
Sharks
Rays
Mollusks
Crustaceans
5
MULTIMARKET (MM)
17%
17%
66%
Bony fish
Sharks
Mollusks
6
RESTAURANTS (RT)
4%
27%
60%
2%
7%
Bony fish
Sharks
Devil Rays
Mollusks
Crustaceans
