# Supplementary material for: A glimpse into the genetic diversity of the Peruvian seafood sector: Unveiling species substitution, mislabeling and trade of threatened species
Source: PLoS One. 2018 Nov 16;13(11):e0206596. doi: 10.1371/journal.pone.0206596 (PMC6239289; doi:10.1371/journal.pone.0206596)
Supplement: S1 Appendix — (PDF) [file pone.0206596.s006.pdf]

S1 Appendix

Phylogenetic identification results of samples SF65 striped marlin *Kajikia audax* and SF131 Atlantic white marlin *K. albida*

Sample SF65 (labeled as marlin) collected from FLS (Tumbes) was landed as a whole body, whereas sample SF131 was a frozen filet collected in an MM (Lima) and sold as sailfish. Identification of sample SF65 based on COI using the BOLD database provided best match values with Indo-Pacific striped marlin *Kajikia audax* (100% similarity) and Atlantic white marlin *K. albida* (99.85% similarity). Also, identification of sample SF131 resulted in 100% and 99.85% similarity with *K. albida* and *K. audax* respectively. The analyses of different mitochondrial and nuclear markers have suggested a high genetic affinity between both species [1, 2], which are believed to have diverged very recently [2, 3, 4]. This limited genetic differentiation may also suggests that both species comprise two allopatric subspecies with some degree of gene flow occurring at points of contact [2, 4], or might simply be different populations of the same species [2]. However, a potential genetic separation between both species was noted in the sequence analysis of the variable mtDNA control region [1, 2]. Based on that evidence, we successfully amplified the complete mitochondrial control region from samples SF65 and SF131 to perform a phylogenetic identification analysis that included 719 homologous sequences (*K. albida* n=188, *K. audax* n=531) gathered from GenBank. Both phylogenetic analyses (BI and NJ, Fig A) showed similar results with all *K. albida* specimens clustered into a single clade (Bayesian posterior probability 51%, NJ bootstrap support 70%) with a mean intraspecific divergence of 3.2% (K2P), whereas *K. audax* sequences formed two independent groups: a "small" group (n=71, Bayesian posterior probability 93%, NJ bootstrap support 100%) with a mean within-cluster divergence of 0.8% (K2P), and a "large" group (n=460, Bayesian posterior probability 56%, NJ

bootstrap support 89%) showing a mean within-cluster divergence of 3.6% (K2P). Sample SF65 was placed within the "small" *K. audax* group, while sample SF131 was within the *K. albida* clade (Fig A). The "small" *K. audax* group showed mean genetic divergences (K2P) of 6.5% and 7.5% to *K. albida* clade and the "large" *K. audax* group, respectively. Similar results were described by Hanner et al. [2] (based on the analysis of 170 control region sequences: *K. audax* n=79, *K. albida* n=91) in which most of the sequences clustered in two separate clades by species, however a small *K. audax* group (n=5) was clustered in a third separate clade with greater similarity to the *K. albida* clade. Only one *K. audax* sequence (GenBank accession DQ199998) was clustered within the *K. albida* clade (Fig A). Further studies using more molecular markers are needed to elucidate the existence of natural hybrids between the closely related *K. albida* and *K. audax*. Based on our phylogenetic analysis results, we assigned sample SF65 (landed as a whole body) to striped marlin *K. audax*, and sample SF131 (frozen filet) to Atlantic white marlin *K. albida*.

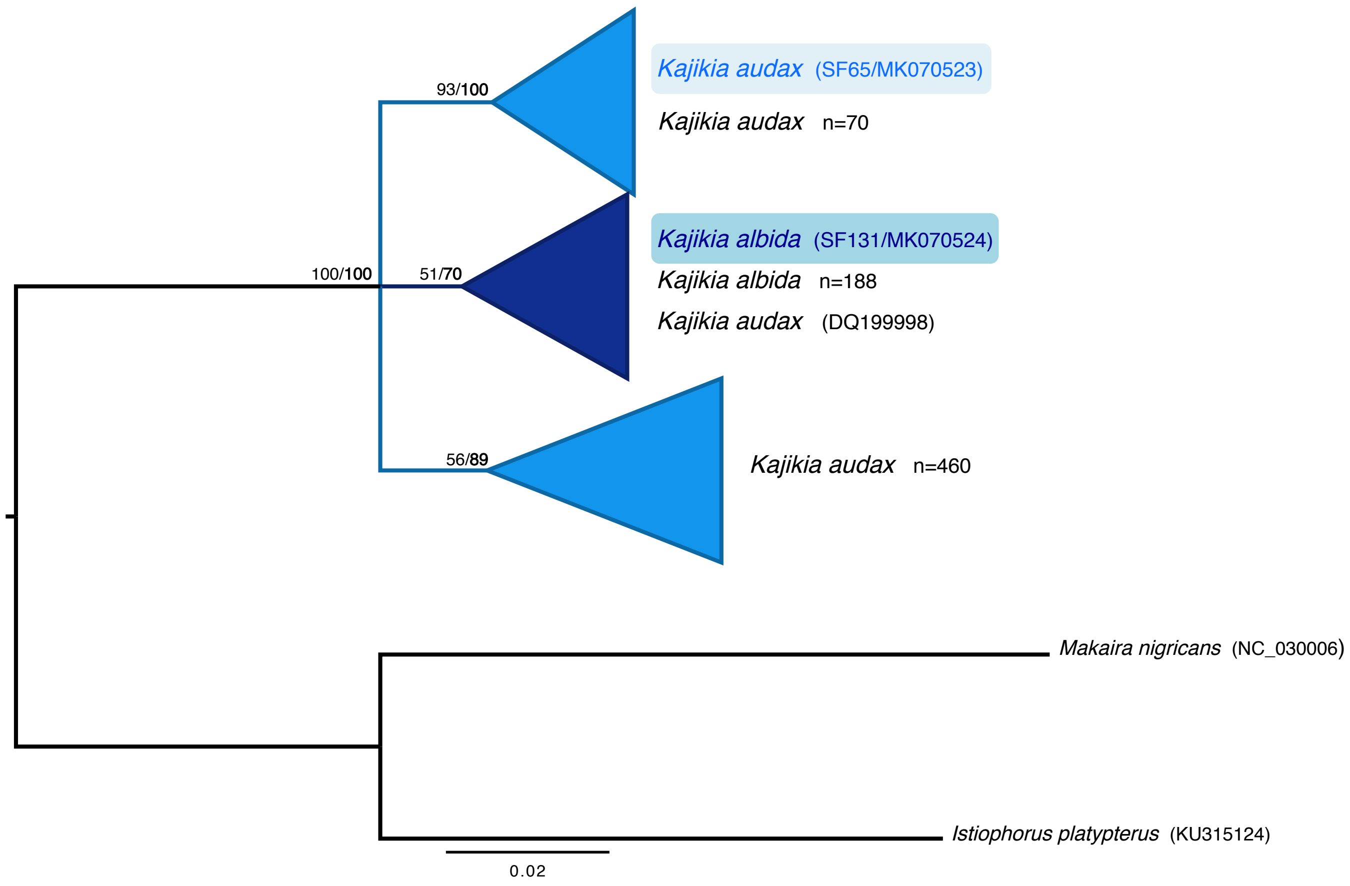

Fig A. Phylogenetic tree based on Bayesian inference (BI) and Neighbor-Joining (NJ) for the identification of samples SF65 *Kajikia audax* and SF131 *K. albida*. Phylogenetic tree based on mitochondrial control region sequences (final matrix 646 bp) from samples SF65 and SF131 (this study) and *K. audax* and *K. albida* homologous sequences available in GenBank. Bayesian consensus tree was inferred with three million generations under the HKY+G substitution model. NJ tree was constructed with 1000 bootstrap replicates under the Kimura-2-parameter (K2P) model. Nodal supports for Bayesian inference posterior probabilities and bootstrap values for NJ analysis (highlighted in bold) above 50% are shown. Samples from this study include identification codes and GenBank accession numbers. The collapsed branches formed by *K. audax* are highlighted in sky blue and the collapsed branch formed by *K. albida* is

highlighted in blue. Blue marlin *Makaira nigricans* and Indo-Pacific sailfish *Istiophorus platypterus* were used as outgroup.

References

1. Graves JE, McDowell JR. Stock structure of the world's istiophorid billfishes: a genetic perspective. Mar Freshw Res. 2003; 54(4): 287-298.

2. Hanner R., Floyd R., Bernard A, Collette BB, Shivji M. (2011). DNA barcoding of billfishes. Mitochondrial DNA. 2011; 22(1): 27-36.

3. Collette BB, McDowell JR, Graves JE. 2006. Phylogeny of Recent billfishes (Xiphioidei). Bull Mar Sci. 2006; 79: 455–468.

4. Williams SM, McDowell JR, Bennett M, Graves JE, Ovenden JR. Analysis of whole mitochondrial genome sequences increases phylogenetic resolution of istiophorid billfishes. Bull Mar Sci. 2018; 94(1): 73-84.
