# Supplementary material for: A glimpse into the genetic diversity of the Peruvian seafood sector: Unveiling species substitution, mislabeling and trade of threatened species
Source: PLoS One. 2018 Nov 16;13(11):e0206596. doi: 10.1371/journal.pone.0206596 (PMC6239289; doi:10.1371/journal.pone.0206596)
Supplement: S2 Appendix — (PDF) [file pone.0206596.s007.pdf]

S2 Appendix

Phylogenetic identification results of samples SF56, SF57, and SF85 smooth-hound *Mustelus* sp.

Three samples bought from WFM-LL (SF56), SMC-LL (SF57), and MM-LI (SF85) were first molecularly identified as *Mustelus henlei* (BOLD similarity 98.15-98.55%). However, BI and NJ phylogenetic analyses (Fig A) that included other *Mustelus* DNA sequences from the BOLD database clustered SF56, SF57, and SF85 samples in a unique clade (Bayesian posterior probability 100%, NJ bootstrap support 98%) with the shortest congeneric divergences of 1.6% and 1.9% (K2P) to *M. henlei* and *M. canis* clades, respectively. Therefore, those three samples were assigned to *Mustelus* sp. Similar results were described by Velez-Zuazo et al. [1] where the lack of *Mustelus* reference sequences hampered the species identification of three shark samples (assigned also to *Mustelus* sp.) reporting an average genetic distance of 2.1 and 2.3% with *M. henlei* and *M. canis*, respectively. Further sequence comparison and phylogenetic analysis confirmed that samples assigned to *Mustelus* sp. by Velez-Zuazo et al. [1] belong to the same species as ours (X. Velez-Zuazo, personal communication). Sample SF56 was declared as "*tollo mamita*" (local name for *M. whitneyi* in northern Peru) which may suggest that samples SF56, SF57, and SF85 belong to the humpback smooth-hound *M. whitneyi* (this species lack of reference sequence in BOLD). Besides, as discussed in Velez-Zuazo et al. [1], *M. whitneyi* is one of the most commonly landed *Mustelus* species dominating the fishing reports. Although identification of sample SF75 (bought in SMC-LI and declared as smooth-hound) showed no ambiguous results (it was identified as *M. lunulatus* with BOLD similarity 100%), it was included in phylogenetic analysis of *Mustelus* specimens. Phylogenetic results included sample SF75 in the monophyletic *M. lunulatus* group, with Bayesian posterior probability 100% and NJ bootstrap support 99% (Fig A), showing a maximum of 0.9% (K2P) within-cluster divergence.

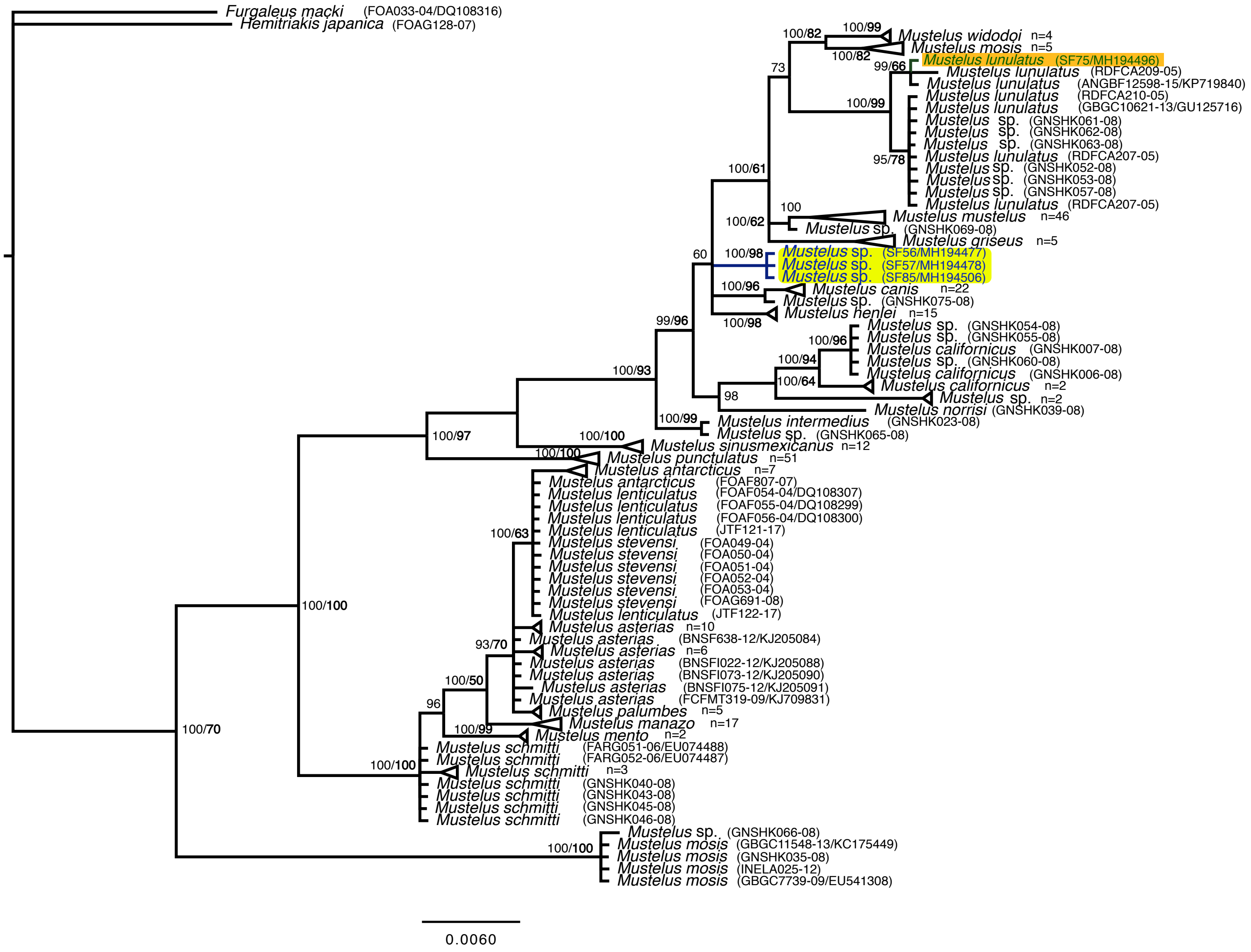

Fig A. Phylogenetic tree for based on Bayesian inference (BI) and Neighbor-Joining (NJ) for the identification of samples SF56, SF57, SF85 *Mustelus* sp. and SF75 *Mustelus lunulatus*. Phylogenetic tree based on COI barcode sequences (final matrix 492 bp) of samples from this study SF56, SF57, SF85 (*Mustelus* sp., highlighted in blue, clade shaded in yellow), SF75 (*M. lunulatus*, highlighted in green and shaded in orange) and other *Mustelus* reference sequences available in BOLD and NCBI. Bayesian consensus tree was inferred with one million generations under the GTR+I+G substitution model. NJ tree was constructed with 1000 bootstrap replicates under the Kimura-2-parameter (K2P) model. Nodal supports for Bayesian inference posterior probabilities and bootstrap values for NJ analysis (highlighted in bold) above 50% are shown. Reference sequence labels include BOLD process ID and GenBank

accession numbers. Whiskery shark *Furgaleus macki* and Japanese topeshark *Hemitriakis japanica* were used as outgroup.

References

1. Velez-Zuazo X, Alfaro-Shigueto J, Mangel J, Papa R, Agnarsson I. What barcode sequencing reveals about the shark fishery in Peru. Fish Res. 2015; 161: 34-41.
