# Supplementary material for: A glimpse into the genetic diversity of the Peruvian seafood sector: Unveiling species substitution, mislabeling and trade of threatened species
Source: PLoS One. 2018 Nov 16;13(11):e0206596. doi: 10.1371/journal.pone.0206596 (PMC6239289; doi:10.1371/journal.pone.0206596)
Supplement: S3 Appendix — (PDF) [file pone.0206596.s008.pdf]

S3 Appendix

Phylogenetic identification results of samples SF42 and SF44 butterfly ray *Gymnura* sp.

Samples SF42 and SF44 collected from FLS were labeled as butterfly ray. The former was bought as fresh filet and the latter was landed as a whole body. Identification of both samples using the BOLD system provided a best match (97.65-97.7% similarity) with *Gymnura marmorata*. However, this result was based on the genetic distance among our query samples and only one reference sequence identified as *G. marmorata* (BOLD process ID PHANT702-08), whereas comparison with three other reference sequences of *G. marmorata* available on BOLD database showed similarity values below 85.8%. Both phylogenetic analyses (BI and NJ, Fig A) showed similar results placing samples SF42 and SF44 in a unique subclade within the clade containing *G. marmorata* (PHANT702-08), which showed a genetic divergence of 2.5% (K2P) to our samples. The other three *G. marmorata* sequences formed a well-supported (Bayesian posterior probability 100%, NJ bootstrap support 100%) monophyletic group, from which the minimum congeneric divergence to our query samples was 16.2% (K2P). Thus, we believe that sequence PHANT702-08 (identified as *G. marmorata*) may represent a cryptic species or a misidentification. The next lowest congeneric genetic distance was detected between our query sample SF42 and the Atlantic smooth butterfly ray *G. micrura* group (14.7%, K2P). There are two reported species from the genus *Gymnura* in Peru: *G. afuerae* (proposed as a junior synonym of *G. crebripunctata* [1]) and *G. marmorata* [2]. To date, only the latter has reference sequences available in public repository, which constrained our sequence comparison only to *G. marmorata*, therefore, samples SF42 and SF44 were assigned to *Gymnura* sp. We cannot rule out the possibility that samples SF42 and SF44 belong to the Peruvian butterfly ray *G. afuerae*.

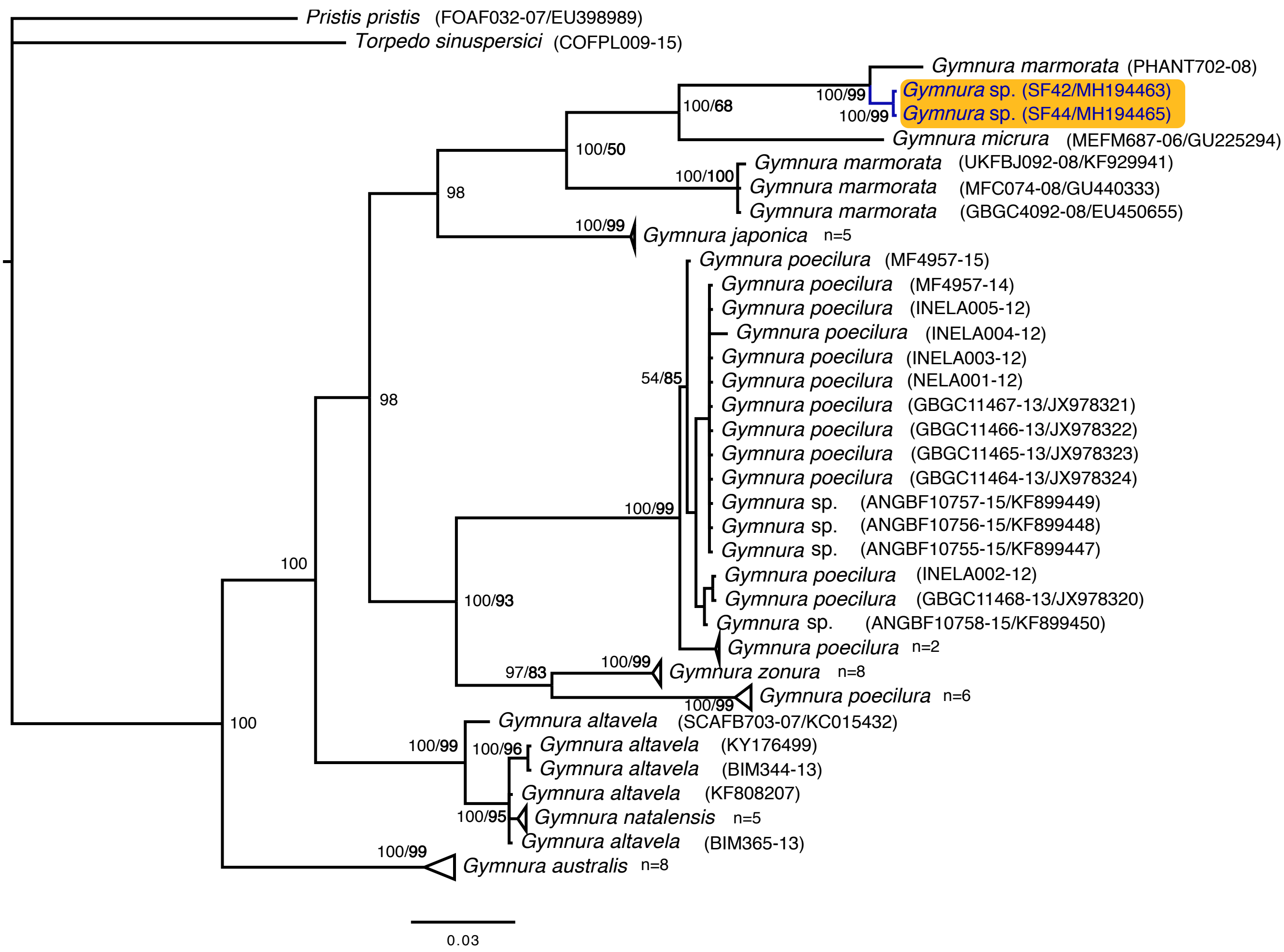

Fig A. Phylogenetic tree based on Bayesian inference (BI) and Neighbor-Joining (NJ) for the identification of samples SF42 and SF44 *Gymnura* sp. Phylogenetic tree based on COI barcode sequences (final matrix 575 bp) from samples SF42 and SF44 (this study) and *Gymnura* reference sequences available in BOLD. Bayesian consensus tree was inferred with one million generations under the HKY+G substitution model. NJ tree was constructed with 1000 bootstrap replicates under the Kimura-2-parameter (K2P) model. Nodal supports for Bayesian inference posterior probabilities and bootstrap values for NJ analysis (highlighted in bold) above 50% are shown. Samples from this study include identification codes and GenBank accession numbers. Reference sequence labels include BOLD process ID and GenBank accession numbers. The branch formed by samples SF42 and SF44 identified as *Gymnura* sp. is highlighted

in blue and shaded in orange. Marbled electric ray *Torpedo sinuspersici* and largetooth sawfish *Pristis pristis* were used as outgroup.

References

1. Last P, White W, de Carvalho M, Séret B, Stehmann M, Naylor G (Eds.). Rays of the World. Csiro Publishing. 2016.

2. Cornejo R, Velez-Zuazo X, Gonzalez-Pestana A, Kouri C, Mucientes GR. An updated checklist of Chondrichthyes from the southeast Pacific off Peru. Check List. 2015; 11(6): 1809.
