# Supplementary material for: A glimpse into the genetic diversity of the Peruvian seafood sector: Unveiling species substitution, mislabeling and trade of threatened species
Source: PLoS One. 2018 Nov 16;13(11):e0206596. doi: 10.1371/journal.pone.0206596 (PMC6239289; doi:10.1371/journal.pone.0206596)
Supplement: S4 Appendix — (PDF) [file pone.0206596.s009.pdf]

S4 Appendix

Conservation status and regulatory framework of sharks

Sharks are considered to be globally under threat, although poorly represented in most fishery management plans [1]. Overfishing, bycatch, and finning driven by the high demand for shark fins in some Asian countries are among the main threats to shark species around the world. Aiming to guarantee a sustainable use of shark (and related elasmobranchs) stocks and to design a shark fisheries management plan, The National Action Plan for the Conservation and Management of Elasmobranchs and Related Species (PAN Tiburón-Perú, DS N° 002-2014-PRODUCE) was published by the Peruvian government in 2014.

There are 66 shark species inhabiting Peruvian waters [2]. According to the "FISHLANDINGS-2017" list, the 10 most commonly landed shark species in Peru during 2017 included six species listed as Vulnerable by IUCN: shortfin mako *Isurus oxyrinchus*, smooth hammerhead *Sphyrna zygaena*, pelagic thresher *Alopias pelagicus*, common thresher shark *A. vulpinus*, humpback smooth-hound *Mustelus whitneyi*, and spotted houndshark *Triakis maculata*. Three species were listed as Near Threatened: blue shark *Prionace glauca*, copper shark *Carcharhinus brachyurus*, and speckled smooth-hound *M. mento*. One species was listed as Data Deficient: redspotted catshark *Schroederichthys chilensis*. In spite of the fact that six of the 10 most landed shark species are listed as "Vulnerable" by the International Union for Conservation of Nature [3], only the smooth hammerhead *S. zygaena* has an established annually fishing and closed season (RM N° 008-2016-PRODUCE) in Peru to date.

A comprehensive description of the regulatory framework related to the Peruvian shark fishery has been described elsewhere [4]. Since then, some important legal provisions have

been added to the framework including the establishment of closed/fishing season and catch limits for the smooth hammerhead *S. zygaena* (RM N° 008-2016-PRODUCE), a law banning the landing and transshipment of shark fins and headless or finless shark carcasses (DS N° 021-2016-PRODUCE), the establishment of authorized landing points and official certificates for shark landings (RD N° 073-2016-PRODUCE/DGSF, RD N° 012-2017-PRODUCE/DGSF, RD N° 019-2018-PRODUCE/DGSFS-PA), and a law banning the capture, landing, transportation, processing and commercialization of whale sharks (RM N° 331-2017-PRODUCE) [5].

Six samples identified as *S. zygaena* were collected during closed season (January 1 to March 10, RM N° 008-2016-PRODUCE). Sample SF66 from FLS-TU was an *S. zygaena* specimen landed as a whole body during closed season, which represents an infraction as stipulated in article 134 numeral 7 of the Regulations on the General Fisheries Act (DS N° 012-2001-PRODUCE, modified by DS N° 017-2017-PRODUCE). This offense is classified as a serious infringement by the Code 7 of Table of Sanctions from the Control Regulation and Sanction of the Fishing and Aquaculture Activities (DS N° 017-2017-PRODUCE). Accordingly, it must be sanctioned by forfeiture of the prohibited fishery products and with a monetary penalty. The other five *S. zygaena* samples detected during closed season (SF50 to SF54) were sold headless and declared as thresher sharks (*Alopias* spp.) at WFM-LL. This seems to be a deliberate case of species substitution and illegal fishing. In Peru the transportation, commercialization or storage of any aquatic resource during closed season constitute an infraction stipulated in the article 134, numeral 75 of the Regulations on the General Fisheries Act (DS N° 012-2001-PRODUCE, modified by DS N° 017-2017-PRODUCE), and shall be sanctioned by forfeiture of the prohibited fishery products and with a monetary penalty according to the Code 75 of the Table of Sanctions from the Control Regulation and Sanction of the Fishing and Aquaculture Activities (DS N° 017-2017-PRODUCE).

Whale sharks

Whale shark *Rhincodon typus* is currently listed as "Endangered" by the IUCN Red List of Threatened Species, listed in Appendix II of Convention on International Trade in Endangered Species of Wild Fauna and Flora (CITES, of which Peru is member since 1975, DL Nº 21080), in Appendix II of the Bonn Convention for the Conservation of Migratory Species of Wild Animals (CMS, Peru ratified this Convention in 1997), in Annex I (Highly Migratory Species) of the UN Convention on the Law of the Sea (UNCLOS, Peru never ratified this Convention), and in Annex I of the Memorandum of Understanding on the Conservation of Migratory Sharks (CMS Sharks MOU, Peru is listed as Range State).

In Peru, the common and brutal finning activity, which consists of removing the fins as the shark is still alive and then throwing the defenseless animal back into the ocean till bleeding to death or to be eaten by other animals, happens offshore as isolated events for whale sharks. For instance, in 2016 a finning incident of a whale shark specimen happened in Acapulco beach (Tumbes) as reported by local news [6]. Favorably, the Peruvian government has taken actions by banning landings and transshipment of shark fins and headless or finless shark carcasses since 2016 (Supreme Decree Nº 021-2016). It is noteworthy that since July 15, 2017, the Ministry of Production of Peru (PRODUCE) enacted the Ministerial Resolution RM N° 331-2017-PRODUCE [5] banning the capture, landing, transportation, processing, and commercialization of this shark species.

Whale shark landings, as well as isolated events of unreported landings, have been happening in Peru since 2006 [4]. In this study, one *R. typus* specimen (SF46) landed as a whole body was collected in January 2017 from FLS at the same spot (Acapulco beach) where the above-mentioned finning incident took place. The fate of this specimen was the local market. Fortunately, according to local fishermen, whale shark meat is not well accepted by consumers

due to its “watery” taste. Whale sharks tend to like warm waters all over the world, including northern Peru and Ecuador, where they sometimes get entangled in fishing nets. A laudable whale shark rescue (caught on video) by a group of fisherman from Tumbes (north of Peru) who untangled and liberated the trapped animal was reported on August 26, 2016 [7]. The evidence described in this study regarding the anthropogenic threats toward *R. typus* in northern Peru demands immediate and stronger conservation actions. A continuous monitoring of landing spots, removal of “ghost gears” (derelict fishing gears) as well as suitable training for local fisherman and community must be concerted by the government, NGOs, local communities, and private sector.

References

1. Davis B, Worm B. The International Plan of Action for Sharks: How does national implementation measure up?. Mar Policy. 2013, 38: 312-320.
2. Cornejo R, Velez-Zuazo X, Gonzalez-Pestana A, Kouri C, Mucientes GR. An updated checklist of Chondrichthyes from the southeast Pacific off Peru. Check List. 2015; 11(6): 1809.
3. The IUCN Red List of Threatened Species. Version 2017-3. 2017. Available from: http://www.iucnredlist.org Downloaded on 05 December 2017.
4. Gonzalez-Pestana A, Kouri JC, Velez-Zuazo X. Shark fisheries in the Southeast Pacific: A 61-year analysis from Peru. F1000Research. 2016; 3:164.
5. Prohíben la extracción de la especie tiburón ballena en aguas marinas de la jurisdicción Peruana. El Peruano. 13 Jul 2017. Resolución Ministerial Nº 331-2017-PRODUCE. Available from: http://busquedas.elperuano.pe/normaslegales/prohiben-la-extraccion-de-la-especie-tiburon-ballena-en-agu-resolucion-ministerial-n-331-2017-produce-1544007-1/ Accessed 3 May 2018.

6. Pescadores mataron a tiburón ballena solo para quitarle sus aletas. RPP Noticias (Radio Programas del Perú). 19 Feb 2016. Available from: http://rpp.pe/peru/tumbes/pescadores-matan-a-un-tiburon-ballena-para-quitarle-sus-aletas-noticia-939404 Accessed 4 May 2018.

7. Pescadores rescatan a tiburon ballena atrapado en redes. RPP Noticias (Radio Programas del Perú). 25 Aug 2016. Available from: http://rpp.pe/peru/tumbes/tumbes-pescadores-rescatan-a-tiburon-ballena-atrapado-en-redes-noticia-990026 Accessed 4 May 2018.
