# Supplementary material for: A glimpse into the genetic diversity of the Peruvian seafood sector: Unveiling species substitution, mislabeling and trade of threatened species
Source: PLoS One. 2018 Nov 16;13(11):e0206596. doi: 10.1371/journal.pone.0206596 (PMC6239289; doi:10.1371/journal.pone.0206596)
Supplement: S5 Appendix — (PDF) [file pone.0206596.s010.pdf]

S5 Appendix

Conservation status and regulatory framework of mobulids

Devil rays and mantas are sensitive to even moderate levels of fishing pressure because of their low post-release survival and an extremely low reproductive rate [1]. Furthermore, the high demand for gill plates in combination with a largely unmonitored and unregulated catches from target and incidental fisheries around the world [2] make those gentle and harmless animals subject to serial depletion or in the worst-case scenario, biological extinction. With the recent synonymization and subordination of *Mobula japanica*, *M. mobular* (one of the five *Mobula* species that inhabit Peruvian waters) is waiting for a reassessment for the Red List [3]. Of the four remaining species that occur in Peru, two are listed as Vulnerable (*M. tarapacana* and *M. birostris*), and the other two (*M. munkiana* and *M. thurstoni*) as Near Threatened by the IUCN. Eleven *Mobula* species, comprising only eight accepted species and three considered to be junior synonyms [4] are listed in Appendix II of CITES (since October 2016) and Appendixes I and II of Convention for the Conservation of Migratory Species of Wild Animals (CMS).

In this study, we have detected 10 *M. mobular* samples collected from FLSs, MKs, and RT in three different departments (TU, LA, and LL). No other *Mobula* species were found, which may suggest that among the 5 *Mobula* species, *M. mobular* is subject to a high fishing pressure, which in turn may lead to severe biomass depletion. Peru is among the top five *M. mobular* and *M. tarapacana* fishing countries with Sri Lanka, India, Indonesia and China, accounting an estimated 95% of mobulids global catch. A downward trend in *Mobula* spp. landings from northern Peru has been reported [5] (and references therein). Similar declines have been

recorded in *Mobula* species from Indo-Pacific, Indian and Atlantic Oceans, and the Mediterranean, which may indicate serial depletions through overfishing [5].

Most scientific research, conservation campaigns and legal measures concerning *Mobula* species have been focused on manta rays (i.e. *M. birostris*), despite similar threats across all mobulids [2]. Peru is not an exception; *M. birostris* is currently the only regulated species (RM N° 441-2015-PRODUCE). The five *Mobula* species that inhabit Peruvian waters are listed in The National Action Plan for the Conservation and Management of Elasmobranchs and Related Species (PAN Tiburon-Peru, DS N° 002-2014-PRODUCE) [6]. Despite these efforts, to date, there are no specific regulations for *M. mobular*. In order to obtain vital information for the implementation of concrete conservation methods assuring a proper management of *Mobula* species, further comprehensive studies including population dynamics, genetic diversity, and migration patterns, combined with the correct species identification by using molecular tools have been suggested [6]. In this study, we have shown the efficacy of both full and mini-DNA barcodes in identifying nine *M. mobular* specimens covering whole body, filet, fin, and dried presentations by FB (all with 100% similarity, BOLD and NCBI databases) and one mislabeled restaurant sample (SF18) served as "ray tortilla" (Spanish omelet) by MB approach (BOLD similarity 100%). In addition, the protective epidermal mucus layer coating *Mobula* bodies has proved to be an effective source of amplifiable DNA, which can be successfully used as non-invasive sampling method [7] when dealing with protected living specimens.

References

1. Pardo SA, Walls RHL, Bigman JS. *Mobula tarapacana* (errata version published in 2016). The IUCN Red List of Threatened Species 2016: e.T60199A121705844.

2. Lawson JM, Fordham SV, O'Malley MP, Davidson LN, Walls RH, Heupel MR, et al. Sympathy for the devil: a conservation strategy for devil and manta rays. PeerJ. 2017; 5: e3027.

3. Kyne PM. It's All in the Name: Shark Systematics and the IUCN Red List. IUCN SSC Shark Specialist Group Global Shark Trends Project 2018-2020. 2018. Available from: http://www.iucnssg.org/shark-systematics-and-the-iucn-red-list.html Accessed 21 August 2018.

4. Last P, White W, de Carvalho M, Séret B, Stehmann M, Naylor G (Eds.). Rays of the World. Csiro Publishing. 2016.

5. Convention on the International Trade in Endangered Species of Wild Fauna and Flora (CITES). Response to the notification to the parties Nº 2015/027. Request for new information on fishery management measures for sharks. Information submitted by Fiji. AC28 Inf. 36. 2015. Available from: https://cites.org/sites/default/files/eng/com/ac/28/Inf/E-AC28-Inf-36.pdf Accessed 3 May 2018.

6. Alfaro-Cordova E, Del Solar A, Alfaro-Shigueto J, Mangel J, Diaz B, Carrillo O, Sarmiento D. Captures of manta and devil rays by small-scale gillnet fisheries in northern Peru. Fish Res. 2017, 195: 28-36.

7. Kashiwagi T, Maxwell EA, Marshall AD, Christensen AB. Evaluating manta ray mucus as an alternative DNA source for population genetics study: underwater-sampling, dry-storage and PCR success. PeerJ. 2015; 3: e1188.
