# Supplementary material for: A glimpse into the genetic diversity of the Peruvian seafood sector: Unveiling species substitution, mislabeling and trade of threatened species
Source: PLoS One. 2018 Nov 16;13(11):e0206596. doi: 10.1371/journal.pone.0206596 (PMC6239289; doi:10.1371/journal.pone.0206596)
Supplement: S6 Appendix — (PDF) [file pone.0206596.s011.pdf]

S6 Appendix

Conservation status and regulatory framework of istiophorids

Since 2008, Peru has banned the commercial harvest and commercialization of large pelagic billfish including the Indo-Pacific blue marlin (*Makaira mazara*), black marlin (*Istiompax indica*), striped marlin (*Kajikia audax*), and the Indo-Pacific sailfish (*Istiophorus platypterus*) (DS N° 009-2008-PRODUCE). The only exception corresponds to the recreational billfish fishery which is catch and release [1]. As demonstrated in this study, not only native marlin species are commercialized in Peru, since sample SF131 (MM-LI) was identified as the highly migratory Atlantic white marlin *K. albida*. The five aforementioned species are included in Annex I (Highly Migratory Species) of the UN Convention on the Law of the Sea (UNCLOS, Peru never ratified this Convention). According to the IUCN list, *M. mazara* and *K. albida* are listed as Vulnerable, *K. audax* as Near Threatened, *I. platypterus* as Least Concern, and *I. indica* is listed in the category of Data Deficient.

Herein, we collected one sample (SF65) identified as striped marlin *K. audax*, landed as a whole body in FLS at Cancas (TU). The extraction of the striped marlin violates the article 1 of the Supreme Decree N° 009-2008-PRODUCE. The *K. audax* specimen was landed after the billfish fishing ban came into effect, which constitutes an infraction stipulated in the article 134, numeral 9 of the Regulations on the General Fisheries Act (DS N° 012-2001-PRODUCE, modified by DS N° 017-2017-PRODUCE). Its subsequent commercialization represents another infraction mentioned in the article 134, numeral 74 of the Regulations on the General Fisheries Act (DS N° 012-2001-PRODUCE, modified by DS N° 017-2017-PRODUCE), and should be sanctioned by forfeiture of the prohibited fishery products and with a monetary penalty as stipulated by the

Code 9 of the Table of Sanctions from the Control Regulation and Sanction of the Fishing and Aquaculture Activities (DS N° 017-2017-PRODUCE).

References

1. Correo. Extraeran especies solo para pesca deportiva. 23 Oct 2014. Available from: https://diariocorreo.pe/peru/extraeran-especies-solo-para-pesca-deportiva-292028/ Accessed 21 August 2018.
