# Supplementary material for: A glimpse into the genetic diversity of the Peruvian seafood sector: Unveiling species substitution, mislabeling and trade of threatened species
Source: PLoS One. 2018 Nov 16;13(11):e0206596. doi: 10.1371/journal.pone.0206596 (PMC6239289; doi:10.1371/journal.pone.0206596)
Supplement: S7 Appendix — (PDF) [file pone.0206596.s012.pdf]

S7 Appendix

Regulatory framework related to labeling of manufactured products

In Peru, labeling of manufactured industrial products (domestic or imported) must be in compliance with the Legislative Decree Nº 1304 (29 December 2016) and the regulations published under the authority of this law. The National Institute for the Defense of Competition and the Protection of Intellectual Property (INDECOPI) is the competent authority that monitors labeling requirements at the retail and wholesale level [1]. The Peruvian Technical Standard NTP 209.038:2009 (Revised in 2014, based on CODEX STAN 1-1985) establishes that all packaged food intended for human consumption must carry labels showing the name of the food product, ingredient list, processing aids, net content and drained weight, address of exporter, importer or distributor, country of origin, lot identification, expiry date, storage conditions, sanitary registration, among other requirements. Original labels of imported food products written in foreign languages must be properly translated into Spanish and displayed using an adhesive label.

Labels from all packed goods analyzed herein met all the requirements stipulated in The Peruvian Technical Standard NTP 209.038:2009, except for two vacuum-packed filets, in which only lot number, expiry date, and net content were shown. Our results showed that five (29%) of the 17 analyzed packed samples (six canned, one precooked, four vacuum-packed, four burgers, and two frozen) did not show a scientific name on their labels. All imported products (one shrimp noodle from the USA, two canned Atlantic sardines produced in Morocco and Spain, one MSC certified canned herring from Germany, and two basa filets from Vietnam) showed the country of origin on their labels, except for three filet products (salmon, marlin,

and basa) that were repacked by the retailer staff using new labels that displayed less detailed information than original ones.

References

1. Gestión. ¿Qué debe incluir el etiquetado de un producto?. 31 Dec 2016. Available from: https://gestion.pe/economia/debe-incluir-etiquetado-producto-125650 Accessed 3 May 2018.
